# Smart home-assisted anomaly detection system for older adults: a deep learning approach with a comprehensive set of daily activities

**DOI:** 10.1007/s11517-025-03308-y

**Published:** 2025-01-31

**Authors:** Ander Cejudo, Andoni Beristain, Aitor Almeida, Kristin Rebescher, Cristina Martín, Iván Macía

**Affiliations:** 1 Fundación Vicomtech, Basque Research and Technology Alliance (BRTA), Mikeletegi 57, 20009 Donostia-San Sebastián, Spain; 2 e-Health Department, Biodonostia Health Research Institute, Paseo Dr Begiristain s/n, 20014 San Sebastián, Spain; 3 Faculty of Engineering, University of Deusto, Avda. Universidades, 24, 48007 Bilbao, Spain; 4 Computational Intelligence Group, Computer Science Faculty, University of the Basque Country, UPV/EHU, Leioa, Spain

**Keywords:** Anomaly, Clustering, Deep learning, Older adult

## Abstract

**Abstract:**

Smart homes have the potential to enable remote monitoring of the health and well-being of older adults, leading to improved health outcomes and increased independence. However, current approaches only consider a limited set of daily activities and do not combine data from individuals. In this work, we propose the use of deep learning techniques to model behavior at the population level and detect significant deviations (i.e., anomalies) while taking into account the whole set of daily activities (41). We detect and visualize daily routine patterns, train a set of recurrent neural networks for behavior modelling with next-day prediction, and model errors with a normal distribution to identify significant deviations while considering the temporal component. Clustering of daily routines achieves a silhouette score of 0.18 and the best model obtains a mean squared error in next day routine prediction of 4.38%. The mean number of deviated activities for the anomalies in the train and test set are 3.6 and 3.0, respectively, with more than 60% of anomalies involving three or more deviated activities in the test set. The methodology is scalable and can incorporate additional activities into the analysis.

**Graphical abstract:**

A comprehensive activity monitoring and anomaly detection system for older adults, using sensor data, predictive modeling, and statistical analysis to alert health professionals of irregular behaviors.
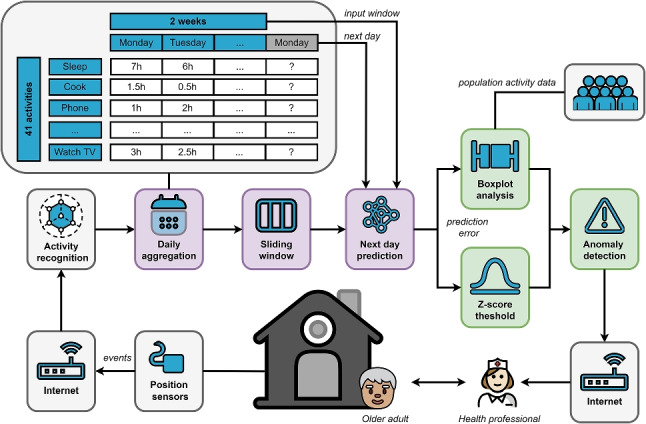

**Supplementary Information:**

The online version contains supplementary material available at 10.1007/s11517-025-03308-y.

## Introduction

The World Health Organization (WHO) states that the proportion of people aged 60 and older is expected to double by 2050, rising from 12 to 24%. The United Nations (UN) has declared 2021–2030 the UN Decade of Healthy Aging and the WHO is leading the initiative to improve the lives of the aging population through global collaboration. One of the primary goals is to provide older adults with quality long-term care [36].

As the population ages, the probability of suffering health problems increases. Chronic illnesses such as cancer and diabetes account for 70% of deaths in the United States and impose significant limitations on daily activities. This health condition lasts for at least one year, requires continuous medical attention, and can limit daily activities. In addition, the 75% of the costs in the United States health system are due to chronic diseases [42]. Other health problems include depression and dementia, which directly affects how a person feels and acts. Risk factors like poor sleep, smoking, and excessive alcohol consumption significantly increase the likelihood of chronic diseases and dementia [34]. With the onset of certain diseases, user’s behavior may be affected due to the inability to perform the same activities or altered dedication to them. An example may be insomnia and lack of energy caused by depression [53]. Detecting significant deviations in user behavior due to changes in health conditions can aid in the early identification of impairments or diseases.

The way individuals engage in and perform daily activities can influence their risk of developing certain health conditions. These activities are categorized into two groups: activities of daily living (ADLs) and instrumental ADLs (iADLs). ADLs include basic tasks essential for survival and well-being, such as bathing, toileting, and feeding. In contrast, iADLs involve more complex activities that support independent living, such as using a mobile phone or managing medications. Evaluating ADLs and iADLs offers critical insights into predicting mild cognitive impairment, dementia, and mortality in older adults. Tools like the Barthel Index and the Lawton Instrumental Activities of Daily Living Scale are used to evaluate both categories [38].

Wearables, home sensors, and mobile phones enable remote and continuous monitoring. The information collected from these devices is non-intrusive, and it is used for activity learning from sensor data [6, 16] and then, for identification of trends and anomalies [19] and automated clinical diagnoses [17], among others [18]. The identification of behavior changes and the development of automatic methods can ease the burden on healthcare professionals and improve the quality of care. Additionally, these technologies can offer critical support to family caregivers, enhancing care efficiency and quality. The availability of data is providing opportunities for the development of new methods and big data software stacks can handle the data obtained from continuous monitoring. The data is used to feed algorithms borrowed from the Artificial Intelligence (AI) field so user-related patterns can be modelled. The growth of the deep learning field in AI benefits from large amounts of data which is generated from remote monitoring and some AI algorithms make use of all the computational power in order to provide fast analysis.

Many state-of-the-art studies analyze the detected activities from smart home sensor data for anomaly detection and behavior modelling of older adults to automatically detect health-related events. However, many activities included in both ADLs and iADLs groups are excluded from the analysis implying a significant gap in current anomaly detection approaches. In addition, recent advances in the field of AI such as deep learning techniques allow the development of methods capable of analyzing all the available activities and not a reduced set of them in a scalable manner [3, 5, 45, 52]. In this study, we advance the state of the art by proposing a double-step anomaly detection system that considers a comprehensive set of activities, specifically 41 activities. This number represents a significant increase compared to previous works, which consider at most 14 different activities. Our methodology is designed to make the detected anomalies interpretable, enabling us to identify activities that deviate from normal behavior.

Therefore, the main objective of this work is to design a system capable of learning user behavior based on the daily dedication of 41 activities in order to detect any significant deviation. For that, AI and more specifically, deep learning methods will be used and compared. The resulting system would have implications in the detection as well as the identification of any alteration that could be related to the onset of a new health condition. Insights of a daily routine and user behavior are also provided with visual analysis and the results of pattern recognition.

The structure of the paper is organized as follows. In Section 2, we delve into the previous research conducted on the analysis of daily activities and anomaly detection. Section 3, provides an explanation of the dataset utilized and the pre-processing steps taken. Section 4 outlines the formulation and rationale behind the proposed techniques. Section 5 presents the quantitative results obtained from the experiments. Section 6 offers a comprehensive discussion, while Section 7 concludes the study by summarizing the key findings.

**Table 1:** Summary of previous works that perform anomaly detection for activities registered with sensor devices

Paper	Dataset	Method	# Activities	# Users	Performance	Evalution
[33]	Aruba/Kyoto from CASAS	ConvLSTM autoencoder	11/5	1/20	F-score 0.12 for repetition, 0.095 for disturbance and 0.688 for confusion	Simulated anomalies
[5]	Van Kasteren	Recurrent neural network	9	3	Accuracy of 89.7%	Simulated anomalies
[8]	Own dataset	Contextual matrix profile	5	15	Recall of 84.3%	Users with dementia with at least one clinically validated event
[32]	Student Life	Jaccard coefficient	3	33	–	No ground truth, authors evaluate behavioral changes
[3]	Aruba/Cairo from CASAS	Neural network, autoencoder and LSTM	11/13	1/4	Accuracy more than 90% in anomaly detection	Ground truth is approximated via boxplot analysis
[25]	HH111 from CASAS/Own	Multi-scale fuzzy entropy	1	1/1	Accuracy of 100%	Anomalies manually labeled by authors
[37]	Simulated	Fuzzy rule-based system	7	1	Accuracy of 95%	Simulated anomalies
[52]	HH111 from CASAS/Own	Consensus Novelty Detection Ensemble	1/1	1/1	Accuracy of 95.97% for HH111 and 98.59% for own data	Simulated anomalies
[30]	Autopsy of Milan, Italy	–	–	–	—	Design of anomaly detection system to detect falls and deaths with PIR sensors, among others
[20]	CASAS	Isudra	14	5	Gmean of 0.0468 for fall	Anomalies from clinical health events
[45]	CASAS	sw-PCAR and virtual classifier	10	2	Behavior change detection in both weeks with a significant health event	Anomalies from clinical health events
[23]	Aruba/Milan from CASAS	Autoencoder	11/15	1/1	More than 90% accuracy for 9	Ground truth is approximated via boxplot analysis

## Related work

There are several works that have performed anomaly detection (see Table 1) from smart home sensor data to address specific health issues such as dementia. The Aruba and Kyoto projects from the Center for Advanced Studies in Adaptive Systems (CASAS) dataset [18] were used to analyze dementia-related anomalies using a convolutional long short-term memory (ConvLSTM) deep learning model [33]. The authors fed the model with the sensor readings and their location in the smart home to predict either repetition, disturbance in sleep, or confusion. The study reported F-scores [40] of 0.12 for repetition, 0.095 for sleep disturbance, and 0.688 for confusion, reflecting the performance in each aspect of the model. The authors of [5], used features extracted from sensor data of three different households with windows sizes of 60 s. The data were input into a recurrent neural network (RNN) [31] with 9 different activities to predict whether older adults forgot or repeated activities. The model also aimed to identify potential dehydration and sleep disruptions. To evaluate their approach, they introduced artificially generated anomalies as there are no annotated health events in the dataset, reporting an accuracy score of 89.7%. As reported by [8], data from 15 participants with dementia were analyzed using a contextual matrix profile (CMP) [21], to detect urinary tract infections and hospitalizations with anomaly detection. CMP generates a two-dimensional matrix by computing blocks of time segments. Anomaly scores were derived from the distance between current and previous contexts, with significant deviations identified using z-scores. They reported a recall of 84.3% with 32.1 alerts. According to [32], the StudentLife [51] dataset was used to study behavior patterns and the contextual information with a set of 3 activities with the objective of providing valuable information to mental health specialists. The authors concluded that the proposed approach can provide objective information on multi-modal patterns, but as future work, they sought to include more activities in their analysis.

Other studies have concentrated on general behavioral anomalies rather than specific diseases like dementia. As proposed by [3], they employed data from Aruba and Cairo projects from the CASAS dataset to predict activities from sensor data, detect anomalies, and perform next-activity prediction. Their analysis included up to 13 different activities, significantly fewer than the 41 activities analyzed in our study. An autoencoder was used for anomaly detection, which reconstructs the input data and the reconstruction error (difference between input and output) was considered for anomaly detection. An overall accuracy of 82% was reported for activity detection, 45.4% for next-activity prediction using an LSTM [31], and over 90% for anomaly detection. As presented by [52], the authors proposed an ensemble approach and performed anomaly detection with only a user and the sleep activity with the CASAS HH111 dataset having either 31 or 18 days for training. The anomaly detection was done by means of z-score, but only up to 6 activities were considered and several models were trained. They achieved an accuracy score in anomaly detection of 97% and the authors stated that the normality score can be dynamically adjusted to incorporate changes in human activities. Likewise, as indicated by [25], they analyzed continuous passive infrared (PIR) sensor data in the HH111 dataset with a multi-scale fuzzy entropy method, which computes ambiguity and uncertainty in time series data, to predict if a given activity of daily living (ADL) is normal or abnormal achieving accuracy in anomaly detection of 98% and 100%. The authors of [37] proposed a rule-based anomaly detection system considering up to seven daily activities which are marked for each user with a criticality level depending on how important the activity is for the user. They added the StreamingBandit [29] application for personalized feedback after detecting an anomaly. The accuracy in anomaly detection reported by the authors after simulating both normal and anomalous behavior achieved a 95%.

Some studies focused on identifying anomalies for detection, rather than for prevention, where an anomaly is understood as an event that has an immediate negative impact on the user’s health status. As proposed by [30], an inactivity model was designed where unexpected scenarios are notified as fast as possible such as myocardial infarction and fall. Similarly, [20] proposed three approaches for anomaly detection that are compared for the detection of clinically meaningful health events: supervised, unsupervised, and indirectly supervised. With the health events and sensor data of five homes from the CASAS dataset, they proposed the Isudra algorithm to improve unsupervised anomaly detection methods. The reported results demonstrated improved performance for supervised and unsupervised anomaly detection algorithms.

Finally, there are two studies which have also studied daily routines as the daily dedication to each of the activities. According to [45], the authors presented a behavior change detection (BCD) approach with two case studies from the CASAS dataset and considering up to 9 activities. With prior knowledge of significant health events, they wanted to demonstrate that users’ daily routines before and after the health events can be distinguished. For that, they defined windows of n days where each day includes not only the amount of time spent in each activity but also the sensor density of each activity and the total amount of movement that occurs at home. Thus, they employed a Virtual Classifier for binary classification. Daily routines with clustering have been explained before [14], with data collected from wearable devices and not from smart home environments. The authors considered activity sequences for each period of the day (e.g., morning and afternoon), identifying the most common sequence of activities for each period. The results indicated a routine coverage value applying collaborative clustering of 89.83%.

To the best of our knowledge, existing studies typically select a limited subset of activities (see Table 1), often omitting important ADLs and iADLs such as mobile phone use, medication management, and cooking. These unused activities are employed by many assessment tools such as the previously mentioned Lawton Instrumental Activities of Daily Living Scale, which can result in a lose of information and missed health events related to certain activities, leading to less accurate assessments. In prior research labeled anomalies related to activity dedication are not used. Studies that reported supervised classification metrics, such as accuracy and F1 score, often introduced anomalies manually [5, 25, 33, 37, 52] or inferred them from significant clinical events [8, 19]. Studies predicting clinical events primarily targeted detection tasks (identifying the presence of a health condition) rather than prevention tasks (anticipating the onset of a health condition). While supervised methods have shown potential in capturing behavioral patterns, the anomaly detection approach described here draws inspiration from previous work but has been adapted to meet real-world challenges. These challenges include the lack of professionally labeled anomalies, the need for scalability across diverse activities, and simplified management of AI models.

For this reason, in our study, we want to significantly extend the number of daily activities considered compared to previous works and perform behavior modelling and anomaly detection while considering all the information obtained. In addition, the proposed approach must be scalable to easily include more activities into the analysis, avoiding the management of several models for a single prediction. In this context, deep learning models are well suited, as these are able not only to give as output more than one label (in this case activities) but also to consider the temporal component of the input. Additionally, algorithms and AI models are typically trained and used for a single user, without taking into account different human behaviors that may be common but not directly related to an anomaly. This can further limit the accuracy and usefulness of these models for detecting health events.

## Materials

Our work focuses on the Center for Advanced Studies in Adaptive Systems (CASAS) dataset [18]. Other datasets have also been employed by previous studies to provide complementary information and support related research. For instance, [49] made the Kasteren wireless sensor network dataset available and Ordonez’s dataset was collected by a set of simple state-change sensors installed in five different environments by [35]. Lastly, [47] conducted their research on data collected from two homes [43]. We use the CASAS dataset as it is the only publicly available dataset$^{1}$ among those mentioned. It is anonymized and includes data from smart homes equipped with sensors placed throughout single-resident apartments, where older adults live independently.

**Table 2:** Simplified sample of sensor data in smart homes where each row is a sensor event with the following features: event registration date, spot, value read, and the recognized activity

2012-09-05 15:57:02.608848	Ignore	14	Dress
2012-09-05 15:57:02.665670	Bedroom	OFF	Dress
2012-09-05 15:57:03.428454	Ignore	61	Other_Activity
2012-09-05 15:57:03.488005	Bathroom	ON	Other_Activity
2012-09-05 15:57:04.412168	Bedroom	ON	Other_Activity
2012-09-05 15:57:05.517908	Bedroom	OFF	Other_Activity
2012-09-05 15:57:08.351990	Bathroom	OFF	Other_Activity
2012-09-05 15:57:09.588479	Ignore	60	Other_Activity
2012-09-05 15:57:09.633537	Bathroom	ON	Personal_Hygiene
2012-09-05 15:57:12.763632	Ignore	61	Other_Activity
2012-09-05 15:57:12.796334	Bathroom	OFF	Personal_Hygiene
2012-09-05 15:57:13.339173	Ignore	60	Other_Activity
2012-09-05 15:57:13.405810	Bathroom	ON	Personal_Hygiene

**Fig. 1:**
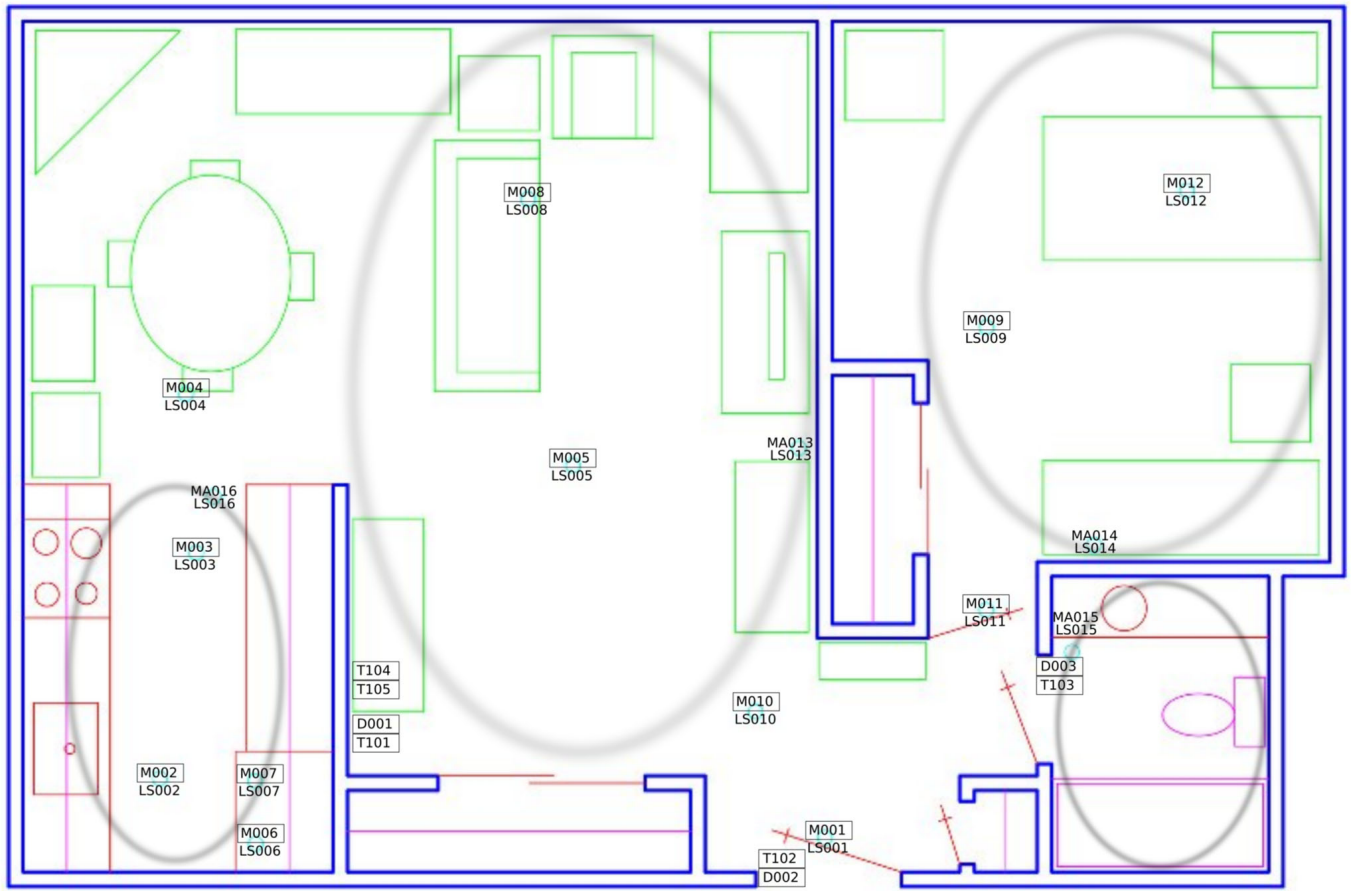
Sensor map of the smart home belonging to user HH101, which is included in the CASAS dataset [18]. Sensors are strategically placed throughout the smart home, each equipped with a unique identifier, providing comprehensive coverage across all areas of the house. For each sensor, an identifier is formed using the type-light sensor (LS), motion sensor (M), door sensor (D), temperature sensor (T), or motion area sensor (MA)-and the sensor number

**Table 3:** Resulting dataset after preprocessing CASAS raw sensor data featured by user and day with the dedication percentage for each activity

user	day	Bathe	Bed_Toilet_Transition	Cook	...
csh101	2012-07-20	0	0	0	..
csh101	2012-07-21	1.03	0	0	..
csh101	2012-07-22	0.23	0.29	0.39	..
...	...	...	...	...	..
csh130	2014-07-13	0.59	0.39	0	..
csh130	2014-07-14	0	0.81	0	..
csh130	2014-07-15	0	0.58	0.2419	..

Considering that the main contribution of this work is to significantly extend the number of activities considered for behavior modelling and anomaly detection, this dataset fits well with the proposed methodology, as 30 users have been monitored, and for each user up to 41 activities have been detected. In Table 2, a small sample of the dataset is shown for a given user. Each row represents an event recorded by a sensor, including the timestamp, sensor details (e.g., location and type), and the recognized activity. The value recorded in an event depends on the sensor type (see Fig. 1). Light sensors capture intensity levels (0 to 100), motion sensors detect movement (ON or OFF), temperature sensors measure ambient temperature, and door sensors record entries (door opened ON or OFF). According to [18], their team can install a new smart home in 2 h and dismantle all the equipment in about 30 min, keeping the costs of installation low. In addition, if a sensor runs low on battery or stops recording events, an alert is sent to the resident, although the battery lasts a year, and it is not frequent to receive one. Once the sensors are installed throughout the home (see an example of sensor distribution in Fig. 1), the authors propose artificial intelligent algorithms to map the most *k* recent sensor registries to a label indicating the activity of the most recent sensor event. In this paper, the authors report an 84% of accuracy in activity recognition with a support vector machine [11] and state that some activities are easier to identify than others. For example, cooking is easy to identify as it has a unique spatio-temporal signature, whereas others are more complicated as they overlap with other activity classes or there is not enough training data. To evaluate the model, activities from 18 smart homes were manually annotated for a one-month period. Fortunately, recent studies focusing on activity recognition have achieved almost 100% of accuracy [4, 13, 27, 28] and provide further information about the sensor types and the analysis for activity recognition.

**Fig. 2:**
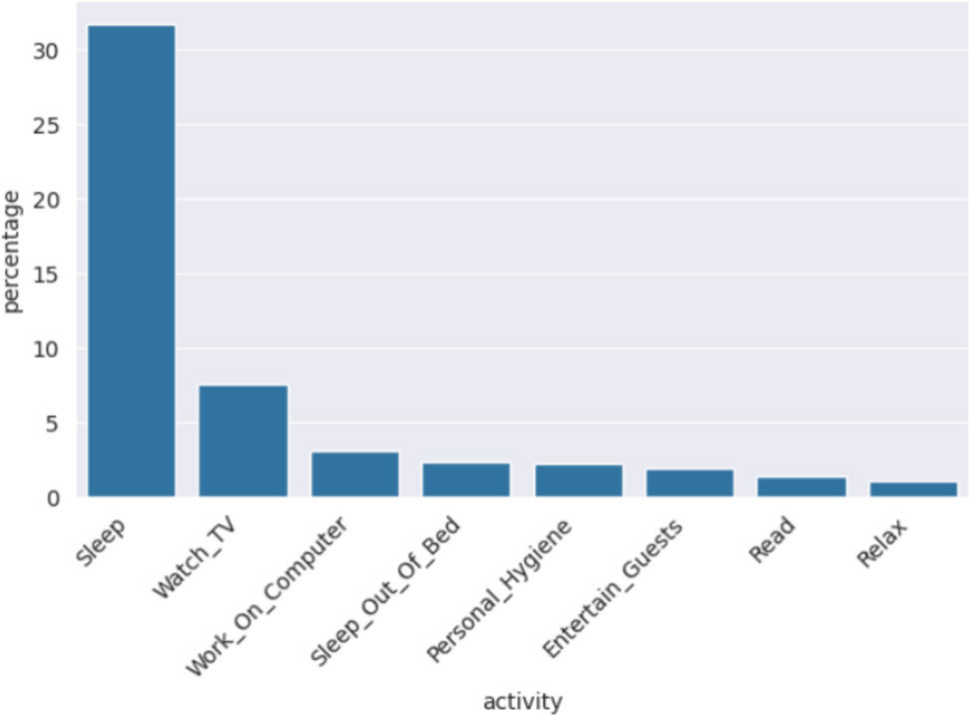
Mean dedication of activities reported in CASAS being the *y*-axis the percentage of the day and *x*-axis some of the different activities

As shown in Table 2, an activity is repeated several times, for that reason, we have merged consecutive activities to determine their start and end times. Then, the activities are framed in a single day and the duration of the activities (i.e., the difference between the end time and start time) is computed. Finally, the same set of activities in a day (e.g., the user might have watched TV three times in a single day) are added up and a day dedication percentage is computed. The result of the preprocessing step is represented in Table 3. If an activity does not appear in a day a 0 is placed.

In Table 3, there are 30 different users with a total of 2,245 days and a mean of 60 registered days per user. The dataset is divided by user, with 70% allocated for training and 30% for evaluation. Thus, both the training and the test subsets contain information of the 30 users but the training subset contains the first 70% of the days for each user and the test set the remaining data.

**Fig. 3:**
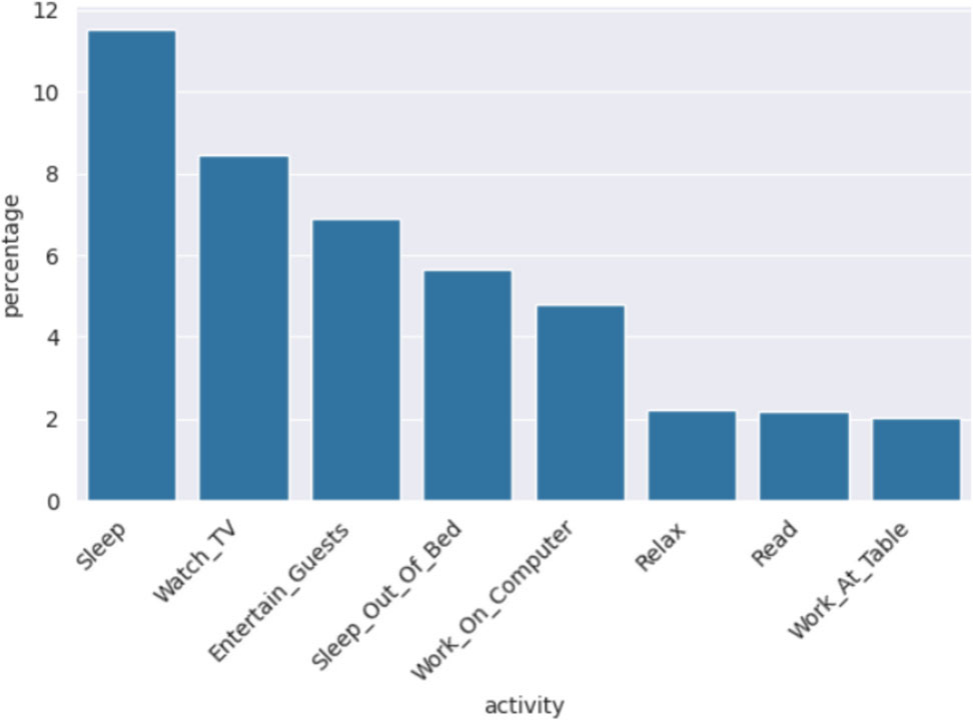
Standard deviation of activities reported in CASAS being the *y*-axis the percentage of the day and *x*-axis some of the different activities

A sliding window of size w (sequence size) is applied to each subset, moving one day forward at each step. The last day in the window is used as the label (value to predict). Finally, a total of 1145 instances (i.e., sequences of days) are used for training and 337 instances are used for testing. Note that for each user the first days can not be estimated (for both train and test sets) as there is not enough previous context to make the prediction. We have not considered splitting the dataset leaving the 70% of the users for training and the rest for testing due to the significant difference in the number of registered days between users, which can bias the proposed models in the training process. In addition, the idea is to learn from the different behaviors and not to be able to generalize with some users to the rest, which is dependent of the target population.

There are up to 41 activities including “Bathe,” “Bed_Toilet_Transition,” “Cook,” “Sleep,” and “Watch_TV,” among others. “Other_Activity” is used for any activity different than the listed ones and we have removed it as it does not have a predictable behavior. Figure 2 shows the overall mean dedication for the first 8 activities with the highest mean whereas Fig. 3 shows the 8 most variable activities. For instance, the mean dedication for “Sleep” is a 31.47% with a standard deviation of 11.54%, a mean and standard deviation of 7.54% and 8.44% respectively for “Watch_TV” and a 1.34% and 2.19% are the mean and the standard deviation for “Read,” respectively.

## Methodology

This section presents the methodology used for user behavior modelling and anomaly detection, with the entire set of activities as input. For each user (see Eq. 1), user *i* is represented as a set of *m* days (see Eq. 2) with the daily dedication percentage per each of the *t* activities (see Eq. 3). We propose methods that utilize registered daily data ($D_{ij}$) from all users (*U*) in the database.

1 $$\begin{aligned} U= &   \{U_{1}, ..., U_{i}, ..., U_{n}\}\end{aligned}$$

2 $$\begin{aligned} U_i= &   \{D_{i1}, ..., D_{ij}, ..., D_{im}\}\end{aligned}$$

3 $$\begin{aligned} D_{ij}= &   \{a_{ij1}, ..., a_{ijh}, ..., a_{ijt}\}, \\ \forall k\in &   \{1,..,t\}, a_{ijk}\in [0,100] \nonumber \end{aligned}$$

### Next-day prediction

Next-day prediction consists of forecasting how a user will behave the next day based only on the previous days. For that, a window (*w*) is set being the first $w - 1$ days the input and the day *w* the label. The input consists of a time series as the days are ordered and the output will depend on how the input is sorted. Each day in the input contains the dedication to all the activities and the output is the expected dedication to each of the activities in the following day.

Next-day prediction is a multi-label regression task, and two groups of models have been considered: ML models and RNN-based models. Among the ML models, the following are included: mean (baseline), KNN [39], support vector machine (SVM) [11], Naive Bayes [24], AdaBoost [41], random forest [7], and Neural Network (Neural_Net) [46]. These algorithms are unable to use more than one window as input, so the temporal component of previous days is not considered, and only the previous day (i.e., a window size of one) is used as input. The Mean is used as a baseline, as it is the simplest method to assign a dedication for each label, with the expectation that any other algorithm will perform better.

For the RNN-based algorithms, the recurrent neural network (RNN), ConvLSTM, RNN with attention (AttentionRNN), and bidirectional RNN (BiRNN) [53] are included (the architecture of each model is depicted in Supplementary Materials, Fig. 5). The RNN serves as the reference model and consists of one or two recurrent layers, where information flows sequentially to the right across the time window. The BiRNN extends this by allowing information to flow both to the right and to the left within the window, enabling the model to capture both past and future temporal dependencies. This capability can be critical for understanding bidirectional influences in activity patterns. The ConvLSTM explicitly integrates a convolutional layer followed by an LSTM-based recurrent layer, enhancing its ability to capture local temporal dependencies while preserving longer-term context. The AttentionRNN comprises two RNN layers with an intermediate attention layer. This attention mechanism computes a set of weights (alpha values) that highlight the most relevant time steps within the window, allowing the model to focus on critical patterns while reducing the impact of less informative time steps. These RNN-based models are designed to process large inputs, with window sizes greater than one, and to capture the temporal evolution of activity dedication. Their inclusion enables a comprehensive evaluation of temporal dynamics and ensures the models can handle varying data complexities and dependencies. These models are compared in Section 5.1 (Tables 4, 5, 6, and 7).

**Table 4:** Results in the test set for the models that have a window (*w*) of size one as input

Model	Window = 1			Window = 1 vs. 15
	MSE	MAE	Time (s)	*p*-value (vs. AttentionRNN)
Mean (Baseline)	8.28 (±1.29)	1.01 (±0.04)	0.00 (±0.00)	0.019
AdaBoost	6.59 (±0.59)	1.21 (±0.04)	0.18 (±0.00)	0.032
Naive Bayes	5.16 (±0.57)	0.78 (±0.02)	0.02 (±0.00)	0.049
KNN	5.55 (±0.52)	0.77 (±0.01)	0.95 (±0.06)	0.027
SVM	8.36 (±1.18)	0.91 (±0.02)	2.38 (±0.49)	0.013
Random forest	5.11 (±0.46)	$\mathbf {0.76 (\pm 0.02)}$	0.04 (±0.00)	0.014
Neural_Net	$\mathbf {4.98 (\pm 0.51)}$	0.81 (±0.05)	0.05 (±0.00)	0.045

The Mean method is considered as a baseline and the best MSE and MAE evaluation metrics are marked in bold. The *p*-value with respect to the best RNN-based model is added by means of paired *t*-test

**Table 5:** Results in the test set varying the window size (i.e., 5 and 10) for different recurrent neural network (RNN) architectures: RNN, ConvLSTM, AttentionRNN, and BiRNN

Model	Window = 5			Window = 10		
	MSE	MAE	Time (s)	MSE	MAE	Time (s)
RNN	4.77 (±0.37)	0.81 (±0.01)	0.33 (±0.03)	5.35 (±0.53)	0.86 (±0.06)	0.20 (±0.02)
ConvLSTM	5.02 (±0.42)	0.83 (±0.01)	0.24 (±0.01)	5.41 (0.54)	0.85 (±0.03)	0.21 (±0.01)
AttentionRNN	5.32 (±0.48)	0.85 (±0.01)	0.34 (±0.09)	7.73 (±2.85)	0.96 (±0.13)	0.35 (±0.02)
BiRNN	4.70 (±0.47)	0.79 (±0.01)	0.62 (±0.04)	5.78 (±1.45)	0.87 (±0.11)	0.64 (±0.05)

The results are measured with three different metrics: mean squared error (MSE), mean absolute error (MAE), and computation time in seconds

**Table 6:** Results in the test set varying the window size (i.e., 15 and 20) for different recurrent neural network (RNN) architectures: RNN, ConvLSTM, AttentionRNN, and BiRNN

Model	Window = 15			Window = 20		
	MSE	MAE	Time (s)	MSE	MAE	Time (s)
RNN	4.76 (±0.47)	0.82 (±0.04)	0.30 (±0.16)	5.48 (±1.78)	0.81 (±0.06)	0.21 (±0.00)
ConvLSTM	5.16 (±0.65)	0.80 (±0.02)	0.34 (±0.00)	5.99 (±1.77)	0.77 (±0.06)	0.70 (±0.54)
AttentionRNN	**4.38 (±0.37)**	0.78 (±0.04)	0.35 (±0.00)	5.33 (±1.86)	0.77 (±0.02)	0.41 (±0.08)
BiRNN	4.48 (±0.49)	0.76 (±0.04)	0.34 (±0.00)	5.28 (±1.85)	**0.73 (±0.02)**	0.36 (±0.01)

The results are measured with three different metrics: mean squared error (MSE), mean absolute error (MAE), and computation time in seconds

The lowest average scores for MAE and MSE in the table are marked in bold

**Table 7:** Results in the test set varying the window size (i.e., 25 and 30) for different recurrent neural network (RNN) architectures: RNN, ConvLSTM, AttentionRNN, and BiRNN

Model	Window = 25			Window = 30		
	MSE	MAE	Time (s)	MSE	MAE	Time (s)
RNN	5.35 (±2.02)	0.76 (±0.07)	0.20 (±0.00)	5.22 (±2.31)	0.75 (±0.04)	0.34 (±0.24)
ConvLSTM	5.75 (±1.88)	0.76 (±0.08)	1.03 (±0.05)	5.97 (±2.58)	0.76 (±0.07)	0.23 (±0.00)
AttentionRNN	6.39 (±2.54)	0.81 (±0.17)	0.40 (±0.09)	7.95 (±2.93)	0.92 (±0.06)	0.35 (±0.03)
BiRNN	5.13 (±2.06)	0.75 (±0.05)	03.4 (±0.00)	5.50 (±2.16)	0.82 (±0.13)	0.66 (±0.00)

The results are measured with three different metrics: mean squared error (MSE), mean absolute error (MAE), and computation time in seconds

In addition, as there are only 30 users and less than 1,200 days for training, we have discarded more advanced approaches such as transformer-based models [50], which benefit from larger amounts of data. Equation 4 shows the formulation of the model where a sequence of $w-1$ days are inputted into a RNN model and the next day ($\hat{D}_{iw})$) is predicted with the estimated dedication for each activity.

4 $$\begin{aligned} f_{RNN} : \mathbb {R}^{(w-1) \times t}&\rightarrow \mathbb {R}^{t} \\ {\textbf {x}} = (D_{i1}, ..., D_{i(w-1)})&\rightarrow f_{RNN}({\textbf {x}})= \hat{D}_{iw} \nonumber \end{aligned}$$

The RNN-based models are composed of long short-term memory (LSTM) [48] cells that are able to capture information in further time steps. The first time step (i.e., day) is introduced inside the first LSTM cell and the output is passed to the next LSTM cell so the second time step can be inputted. This process is repeated with all the time steps (i.e., $w - 1$) until a final output is given. These cells have several neurons inside and all of the cells in the same layer share the same parameters. Finally, a feed-forward layer [46] has been added to match the output with the number of activities (*t*).

For the deep learning models, an additional set of parameters must be specified such as the optimizer function, which defines how the parameters are updated, number of epochs (i.e., number of passes through the entire dataset), dropout (i.e., randomly ignores the specified percentage of neurons from the previous layer to avoid overfitting) and the learning rate. In addition, for the Adam optimizer [10], $\beta _1$ and $\beta _2$ have to be set which are the exponential decay rates for the first and second-moment estimates, respectively. Finally, the batch size is also tuned which defines the number of instances to introduce in the network in each step within an epoch. For each experiment, the optimum number of neurons, learning rate, and dropout are specified while varying the selected architecture and the window size, which are the two settings that have the most significant impact on the next-day prediction performance and therefore, their optimization is crucial.

### Anomaly detection

Once the next-day prediction model is trained, an innovative double-step anomaly detection algorithm is proposed. That is, a day is considered an anomaly when the two following conditions are fulfilled: the error made by the predictive model is over a given threshold and the duration of at least one activity is outside a defined range.

The error is obtained using the mean absolute error (MAE), [12] comparing the predicted day with the actual value after the next-day prediction as in Eq. 4, where the lower the value, the better. Note that other evaluation metrics such as accuracy or F1 are not possible to evaluate as the ground truth of the anomalies is not available in the dataset, and thus, this method relies on the next-day prediction performance of the best RNN-based model. Supervised classification metrics improve the interpretability of results. However, it is not feasible for professionals to label and evaluate the entire sequence of activities, especially when the focus is on the prevention of health conditions (before they occur) rather than detection. Therefore, unsupervised approaches, such as the one proposed for next-day prediction combined with statistical methods like the z-score, appear promising for detecting deviations in a given user’s behavior. Previous studies (see Table 1) using classification metrics either generate synthetic anomalies, approximate ground truth with box-plot analysis, or focus on predicting clinical events (i.e., detection rather than prevention). Additionally, metrics such as MAE allow us to apply z-score anomaly detection to prediction errors in daily activity dedication.

5 $$\begin{aligned} \text {MAE} : (\mathbb {R}^{t}, \mathbb {R}^{t})&\rightarrow \mathbb {R} \\ (D_{iw}, \hat{D}_{iw})&\rightarrow \text {MAE}(D_{iw}, \hat{D}_{iw}) = \epsilon _{iw} \nonumber \end{aligned}$$

After computing the error for all of the days, in the first step, a vector $\varepsilon = \{\{\epsilon _{ij}\}_{i=1}^{n}\}_{j=1}^{m}$ is obtained with all the committed errors by the RNN. Then, a *p*-value is selected so we can get the corresponding z-score (*Z*) in the normal distribution [1] with the mean equal to 0 and a standard deviation of 1. In order to apply the threshold and detect anomalies, each error must be mapped to the z-score in the Gaussian distribution as in Eq. 6. For that, the mean ($\mu$) and the standard deviation ($\sigma$) of all the errors corresponding to every user and day in the dataset have to be computed. The index *p* of the values in the normal distribution can be mapped to *ij* in the error list ($\epsilon$).

6 $$\begin{aligned} \mu&= \frac{\sum _{p=1}^{|\varepsilon |}\epsilon _p}{|\varepsilon |} \\ \sigma&= \sqrt{\frac{\sum _{p=1}^{|\varepsilon |}(\epsilon _p - \mu )^2}{|\varepsilon |}} \nonumber \\ z_{p}&= \frac{e_{p}-\mu }{\sigma }, \forall p \in \{1,..,n*m \} \nonumber \end{aligned}$$

Once the threshold is defined (*Z*) and $z_{p}$ is calculated for each error, an anomaly is detected when $z_{p} > Z$. Notice that *p*-value defines the number of expected anomalies, where using a higher *p*-value would only identify very irregular values as anomalies, reducing the probability of having false positives. For instance, if the selected *p*-value is equal to 0.95, only that 5% of the detected anomalies will correspond to false positives.

In the second step, we compute the boxplot whiskers for each activity (see Eq. 7) in order to explain the anomaly (day) by outputting those activities of the day whose dedication is located in the abnormal area of the boxplot (i.e., under or over the computed upper or lower limit). Q1 is the first quartile which has the 25% of the data behind whereas Q3 is the third quartile and has the 75% of the data behind.

7 $$\begin{aligned} upper_s&= Q3 + 1.5*(Q3_s - Q1_s) \\ lower_s&= Q1 - 1.5*(Q3_s - Q1_s) \nonumber \\ lims&= \{(lower_s, upper_s)\}_{s=1}^{t} \nonumber \end{aligned}$$

In this dataset, there is no feedback regarding the user’s health condition such as questionnaires or comments made by clinicians. For that reason, we combine our approach with a statistical analysis based on variable deviation, considering abnormal a day if at least one activity is outside the common range. Thus, a day ($D_{ij}$) will be considered an anomaly only if $z_{p} > Z$ and if it meets the following requirement: $\exists s(1 \le s \le t \rightarrow a_{ijs} > upper_s \vee a_{ijs} < lower_s )$. With this mechanism that only identifies an event as abnormal if the activity is deviated with respect not only to the model but also to the sample population, we expect that the number of false positives, that could be detected for having increased the number of activities, significantly reduces. Thus, false positives mainly depend on the *p*-value adjusted in the first step and is up to the user the sensitivity the method will have (and therefore the probability of detecting false positives). However, this is a security mechanism, and it can only be evaluated by using true (labeled) anomalies or human feedback.

## Results

In this section, the results are presented and discussed after applying the methods explained in Section 4. In Section 5.1, a model is trained for next-day prediction and behavior modelling. Finally, in Section 5.2, the results of the anomaly detection algorithm are presented.

The clustering and visualization of activity patterns are presented in Supplementary Materials, Section 1, achieving a silhouette score of 0.18 with K-means. Supplementary Materials, Section 2 expands on the results and discussion from Section 5.1 regarding the proposed models for next-day prediction, including the hyperparameters of the best models and an analysis of the predicted values. Additional results and information on the double-step anomaly detection system are provided in Supplementary Materials, Section 3.

### Next-day prediction

In this set of experiments, we aim to measure the capabilities of the different approaches presented in Section 4.1 for next-day prediction based on the previous days of a given user. The objective is to estimate how much time the user will spend on each activity the next day and in this way, the user’s behavior is modeled with all the data provided by a comprehensive set of activities. Having a good performance in next-day prediction for behavior modelling would indicate the ability to model the user activity dedication patterns as well as activity duration dependencies among the different activities. Modelling these two concepts is essential for understanding user behavior and also to generalize across users, as the proposed models are trained with all the users’ data to better understand common behavior in the current population.

For each deep learning model, a set of parameters was adjusted with the Optuna framework [2] in order to optimize the performance of the resulting models in the test set. The hyperparameters adjusted include the number of CNN, LSTM, and FF layers (1–4), the number of filters and kernel size for CNN layers (2–64), the number of neurons in LSTM and FF layers (8–256), and the dropout rate (0–0.4). The number of trials (i.e., combinations of parameters tested by Optuna) is 25. All the proposed models share the same set of parameters for the training step: Adam optimizer, learning rate of 0.1, $\beta _{1}$ set to 0.9, $\beta _{2}$ set to 0.999, mean squared root error (MSE) [12] as the loss function, a batch size of 128, 80 epochs and relu activation function in the last layer as the activity duration is always positive. Keras [15] is the framework used for the implementation of the aforementioned models. For each model, the mean computation time (in seconds) and the standard deviation across three folds are provided. The hardware of the computer where the models have been trained consists of an i9-12900KF CPU and a NVIDIA RTX 4060 GPU. The sklearn models use the CPU, whereas the deep learning models mainly make use of the GPU. As the traditional machine learning models are not suitable for multi-label prediction, a model is trained for each label and the total computation time is calculated as the sum of the prediction time for each label.

Tables 4, 5, 6, and 7 show the results of the different next-day prediction approaches using 3-fold cross-validation. These results are given using mean squared error (MSE), mean absolute error (MAE), and the computation time, being a lower value better for the three metrics. Each of these metrics represents the average score and standard deviation across the three folds, rounded to two decimal places.

In Table 4, the results for the best models with a window size of one are shown. The mean method serves as the baseline for regression, meaning that any other algorithm should perform better. The best score in terms of MSE is achieved by the Neural_Net, with a mean score of 4.98, that is, a relative increase in performance of 39.66% compared to the baseline. In terms of MAE, a mean score of 0.76 is achieved by the random forest, with a relative increase of 24.76% with respect to the baseline.

**Table 8:** Detected anomalies (i.e., abnormal days) with the corresponding user and the activities that have exceeded either the lower or the upper limit whiskers of the activity level boxplot

user	day_id	activity	dedication	description
csh101	8	Watch_TV	54.87%	Dedication over the 29.80%
		Sleep_Out_Of_Bed	11.25%	Dedication over the 3.21%
csh117	40	Sleep_Out_Of_Bed	23.51%	Dedication over the 1.06%
		Bed_Toilet_Transition	1.06%	Dedication over the 0.78%
	252	Entertain_Guests	79.40%	Dedication over the 1.99%
		Sleep	0.03%	Dedication under the 12.66%
csh116	1125	Groom	3.93%	Dedication over the 3.01%
		Entertain_Guests	27.67%	Dedication over the 1.99%
		Cook_Lunch	1.29%	Dedication over the 0.73%

Tables 5, 6, and 7 show that the best mean MSE is achieved by the AttentionRNN for a window size of 15, resulting in a mean score of 4.38 for next day activity dedication prediction. The best MAE is achieved by the BiRNN when the window size is 20. The average of the computational time in seconds across the three folds when predicting all the instances in the test set ranges between 0.2 and 1.03 s for any of the RNN-based models.

A comparison between the best RNN-based model (AttentionRNN) and the Baseline, in terms of mean MSE, shows a relative improvement of 47.19%. When comparing the AttentionRNN with the top-performing model that uses a window size of 1 (Neural_Net), the relative improvement in mean MSE is 12.15%. For each of the models listed in Table 4, the *p*-value from the paired *t*-test is presented. This test compares the MSE scores obtained across the three folds for each model against the AttentionRNN when using a window size of 15. With a threshold of 0.05, the scores achieved by the AttentionRNN show statistical significance with respect to all models considered with a window size of 1. This suggests that considering not only the current day but also the previous days (specifically, the previous two weeks) yields better results. When comparing RNN-based models with a window size of 15, a *p*-value lower than 0.05 was observed for the AttentionRNN compared to both ConvLSTM and RNN models. No statistical significance was observed when comparing the performance of the AttentionRNN with BiRNN.

As a conclusion, the AttentionRNN is considered the best model. Although the best MAE score is achieved by the BiRNN, a better score in MSE is preferred when selecting the model, as this metric penalizes larger errors, which may trigger a higher number of false positives in anomaly detection. Thus, Adding an attention mechanism on top of the RNN has proved to boost the capabilities of the model. This model has improved the mean MSE of the baseline in a 47.19% and the mean MAE in a 32.88%. In addition, when comparing the window sizes, the best results are obtained when *w* is equal to 15, that is, the proposed approaches perform better when taking into account the activities of the previous two weeks and curiously the performance is not improved but slightly worsen when using a one-month window.

### Anomaly detection

In the previous experiment, a model capable of predicting the next day of a user based on the previous two weeks has been developed. In this experiment, we employ this model for anomaly detection considering the error produced by the model. Having a consistent method for anomaly detection can provide an automated system for user monitoring and for the detection of health-related problems with the consideration of a wide range of activities. The *p*-value has been adjusted to 0.925 on a compromise between minimizing the probability of reporting false anomalies and the added value of reporting not such extreme values as anomalies. Previous work has also employed this threshold for anomaly detection [44]. In any case, the threshold depends on the target population. For instance, if anomalies must be detected with even minor deviations (e.g., for fragile populations), the *p*-value should be set to lower thresholds (e.g., 0.85 or 0.9). Conversely, if anomalies are only of interest when behavior deviates significantly from expectations (e.g., in healthy populations), a higher *p*-value (e.g., 0.99) would be more appropriate.

The first step of the proposed anomaly detection system has marked 66 anomalies in the training set and 40 in the test set. In the second step, the number of anomalies has been reduced to 64 and 28 for 17 and 13 users in the train and test set, respectively. Notice that the proposed double-step anomaly detection system has been able to replicate a similar proportion of anomalies in the test set (i.e., unseen data) having almost the 70% of the anomalies in the train set and the 30% in the test set. The mean number of deviated activities for the anomalies in the train and test set are 3.6 and 3.0, respectively, having more than the 60% of the anomalies with 3 or more deviated activities in the test set. This can indicate that, in general terms, the deviation in one activity affects to the dedication of other two activities more.

Table 8 shows four different anomalies (i.e., abnormal days). Within these anomalies, some activities exceed the expected activity dedication. For instance, user “csh101” has spent more than half a day watching TV which is greater dedication than expected when compared to other days. Consequently, this may have had an effect on the time spent sleeping out of bed. User “csh117” on day 40 has spent more time going to bed at night which may be associated with the time spent sleeping out of bed. The same user on day 252 has spent all the day with guests and thus, this user has slept less. Other relations can be found for example on day 1125, where user “csh116” has spent a significant amount of time grooming and cooking which could be related to having guests. Note that these relationships could not have been considered with previous approaches as in this work we significantly increase the number of activities inputted to the next-day prediction model. With these results, we hypothesize that caregivers can be aware of the user’s behavior and detect significant changes. In addition, if the user is under treatment, the anomalies may also indicate behavior that is not conducive to improving the user’s health condition.

The anomaly detection technique presented in this work (see Table 8) can provide some insights. For example, day 8 shows that the time watching TV has increased along with the time sleeping out of bed. According to [22], using media to aid sleep has a negative impact on the sleep quality. Moreover, a repeated and prolonged bad quality of sleep has been related to depressive symptoms, several chronic diseases, and arthritis, among others [26]. Thus, intervention is advisable to prevent health issues. Another example is day 40, as the user sleeps out of bed and makes many trips to the bathroom, which could be related to, for example, nocturia, with a prevalence that increases with age [9]. Similarly, this sleep disorder can be related to depression and with a longer prevalence to an increase of mortality and falls, among others. These two cases are identified by considering a large number of activities as this method supports. Nevertheless, those should be considered as prompt alarm results that should derive into a consultation with a health professional.

As a conclusion, the proposed method is able to automatically report abnormal days without explicitly setting thresholds for each activity. In addition, the anomalies that are detected are conditioned by how the user has been behaving the previous two weeks, and with the results provided in Table 8, an explanation to some of the activities can be found.

## Discussion

Regarding the results, several models based on RNNs have been tested (i.e., RNN, ConvLSTM, AttentionRNN, and BiRNN) with different window sizes (i.e., 5, 10, 15, 20, 25, and 30) and evaluated with 3-fold cross validation leading to an optimal result using mean squared error (MSE) of 4.38% on average for next-day prediction with a window size of 15. With this window size, the input window includes data from two different weekends, as well as repeated occurrences of the same day of the week, not just consecutive days. The results have shown that the model achieves a better performance when looking at the previous two weeks and not when looking to a 5, 10, 20, 25, or 30-day window. Providing more historical data (i.e., larger windows) does not improve the model’s capabilities, and having information of the previous two windows has given better results than taking into account the whole month. This can be down to two reasons: there is enough information in the previous weeks and looking further in the past does not add up relevant information or the model is not able to manage the information provided by Windows with a duration higher than two weeks. The second scenario could be tackled with more sophisticated methods such as transformers [50] to analyze long-term dependencies but more data should also be provided. In addition, the attention mechanism helps the model focus on meaningful information provided by previous days in order to improve the overall performance.

A review of the state-of-the-art has shown that a maximum of 14 activities have been inputted to an anomaly detection algorithm (see Table 1). As in this study, in many cases labeled anomalies are not available and previous works have explored different classification approaches such as the prediction of simulated anomalies [33, 52] or box-plot analysis [3, 23], whereas there are some that predict meaningful clinical events [20, 45]. Thus, a direct comparison with previous works is not feasible, except for the aforementioned comparison in the number of activities considered and their implications. In addition, with anomaly simulation, the proposed method could be biased to a scenario that may not reflect reality. For these reasons, the objective of this study has been to include and evaluate a methodology that can detect anomalies considering a larger number of activities. The results show that some anomalies are explained by activities that have not been considered by any previous work. For example, day 252 in Table 8, which has been marked as an anomaly, shows that the low dedication to sleeping may be explained by a long visit at home. This fact remarks the importance of extending the number of activities considered and opens room to evaluate the proposed methodology with labeled anomalies. In addition, the proposed methodology works regardless of the *t* number, that is, the number of activities considered.

It is important to note that not only the proposed system, but also previous works considering activities detected at home, rely on the precision of the sensors placed throughout the home to model user behavior and detect anomalies. Errors in the readings of the sensor and missed prediction of the activities performed by the user can lead to a shifted interpretation and worse modelling of user behavior, increasing the number of false positives in anomaly detection. Although current algorithms achieve a very good activity recognition performance, detection errors are still present and must be considered when analyzing a detected anomaly. In addition, a limitation of the analysis is the lack of information related to the user such as demographic information or health events during the monitoring period. The availability of this information would help to propose more accurate systems and to better tune the hyperparameters to decrease the number of false positives, as well as avoid missing any significant health event of the user. We acknowledge that the evaluation has not been carried out in a real-world scenario for the prevention of potential health issues but the importance of extending the number of activities considered has been made evident, hoping that future works consider a larger number of activities in real-world settings. As part of our future work, which has not been addressed in this study, we suggest incorporating the order of the activities while still allowing for the analysis of a substantial number of activities. This improvement has the potential to significantly increase the utility of anomalies as they might have different implications depending on the hour of the day that they occur. Finally, having more information would allow the use of more sophisticated algorithms for time series modelling (e.g., transformer-based models) leading to a better user behavior modelling with a decreased next-day prediction error, allowing to develop a more effective anomaly detection system. This would also be beneficial for a comparison of next-day prediction performance when training the proposed system for each individual compared to training the models with all the population data.

The proposed anomaly detection system aims to enhance current healthcare monitoring approaches. It is the first to encompass a wide range of activities, identifying anomalies that include tasks commonly assessed in clinical tools like the Barthel Index and the Lawton Instrumental Activities of Daily Living Scale. By incorporating activities of daily living (ADLs) and instrumental ADLs (iADLs) into the analysis, this system aligns with healthcare professionals’ practices, offering greater precision in the prevention of diverse health conditions.

## Conclusions

In this work, we have applied algorithms borrowed from the deep learning field, along with a clustering technique and statistical methods for the development of an automated system capable of supporting the prevention work done with older adults living in smart home environments. For that, a manual inspection has been carried out with data visualization techniques discovering activity patterns. Then, we have developed an automated system capable of predicting the next day of a user with a recurrent neural network model and a double-step anomaly detection technique. Clustering the daily dedication of 41 activities yields a silhouette score of 0.18. The best-performing model, AttentionRNN, achieves a mean squared error (MSE) of 4.38, representing a 47.19% improvement over the Mean baseline and a 12.15% improvement over the Neural Network. The performance difference between AttentionRNN (with a window size of 15) and all other models, except BiRNN, is statistically significant ($p < 0.05$).

We conclude that our approach is able to effectively model the user’s behavior taking into account a comprehensive set of activities, as the overall errors produced for next-day prediction is around 4% in daily dedication percentage which can be used to automatically detect deviations from the common user behavior or an inappropriate behavior for a given health situation. Moreover, some anomalies could not have been explained without considering the whole set of daily activities. With this work, we open room for research with an extended set of activities and deep learning techniques in order to develop more accurate behavior modelling techniques or anomaly detection systems, by considering different populations with certain health conditions.

We also believe that having more data would be beneficial in order to apply more complex methods such as transformer-based models. The release of demographic data could also provide more insights in order to explain the relationship between the anomalies and the user’s health events. In terms of future work, the anomalies could be used for trend detection and long-term behavior change detection in populations with both higher and lower variability in daily routines.

## Supplementary Information

Below is the link to the electronic supplementary material.

Supplementary file 1 (pdf 1047 KB)

## References

[1] Ahsanullah M, Kibria BM, Shakil M (2014) Normal distribution. In: Normal and student st distributions and their applications, pp 7–50. Springer

[2] Akiba T, Sano S, Yanase T, Ohta T, Koyama M (2019) Optuna: a next-generation hyperparameter optimization framework. In: Proceedings of the 25th ACM SIGKDD international conference on knowledge discovery & data mining, pp 2623–2631

[3] Alaghbari KA, Saad MH Md., Hussain A, Alam MR (2022) Activities recognition, anomaly detection and next activity prediction based on neural networks in smart homes. IEEE Access 10:28219–28232

[4] Aminikhanghahi S, Cook DJ (2019) Enhancing activity recognition using CPD-based activity segmentation. Pervasive Mob Comput 53:75–89 39896094 10.1016/j.pmcj.2019.01.004 PMC11784966

[5] Arifoglu D, Bouchachia A (2017) Activity recognition and abnormal behaviour detection with recurrent neural networks. Proc Comput Sci 110:86–93

[6] Baldominos A, Cervantes A, Saez Y, Isasi P (2019) A comparison of machine learning and deep learning techniques for activity recognition using mobile devices. Sensors 19(3):521 30691177 10.3390/s19030521 PMC6386875

[7] Biau G, Scornet E (2016) A random forest guided tour. Test 25:197–227

[8] Bijlani N, Nilforooshan R, Kouchaki S (2022) An unsupervised data-driven anomaly detection approach for adverse health conditions in people living with dementia: cohort study. JMIR Aging 5(3):e38211 36121687 10.2196/38211 PMC9531007

[9] Bliwise DL, Wagg A, Sand PK (2019) Nocturia: a highly prevalent disorder with multifaceted consequences. Urology 133:3–13 10.1016/j.urology.2019.07.005 31310770

[10] Bock S, Weiß M (2019) A proof of local convergence for the Adam optimizer. In: 2019 International joint conference on neural networks (IJCNN), pp 1–8. IEEE

[11] Cervantes J, Garcia-Lamont F, Rodríguez-Mazahua L, Lopez A (2020) A comprehensive survey on support vector machine classification: applications, challenges and trends. Neurocomputing 408:189–215

[12] Chai T, Draxler RR (2014) Root mean square error (RMSE) or mean absolute error (MAE). Geosci Model Dev Discuss 7(1):1525–1534

[13] Chen L, Nugent CD, Wang H (2012) A knowledge-driven approach to activity recognition in smart homes. IEEE Trans Knowl Data Eng 24(6):961–974

[14] Chifu VR, Pop CB, Rancea AM, Morar A, Cioara T, Antal M, Anghel I (2022) Deep learning, mining, and collaborative clustering to identify flexible daily activities patterns. Sensors (Basel, Switzerland) 22(13) 10.3390/s22134803 PMC9269491 35808297

[15] Chollet F et al (2015) Keras

[16] Cook D (2015) Activity learning: discovering, recognizing, and predicting human behavior from sensor data

[17] Cook D (2015) Activity learning: discovering, recognizing, and predicting human behavior from sensor data

[18] Cook DJ, Crandall AS, Thomas BL, Krishnan NC (2012) Casas: a smart home in a box. Computer 46(7):62–69 10.1109/MC.2012.328 PMC3886862 24415794

[19] Dahmen J, Cook DJ (2021) Indirectly supervised anomaly detection of clinically meaningful health events from smart home data. ACM Trans Intell Syst Technol (TIST) 12(2):1–18 10.1145/3439870 PMC8323613 34336375

[20] Dahmen J, Cook DJ (2021) Indirectly supervised anomaly detection of clinically meaningful health events from smart home data. ACM Trans Intell Syst Technol 12(2):1–18 34336375 10.1145/3439870 PMC8323613

[21] De Paepe D, Hautte SV, Steenwinckel B, De Turck F, Ongenae F, Janssens O, Van Hoecke S (2020) A generalized matrix profile framework with support for contextual series analysis. Eng Appl Artif Intell 90:103487

[22] Exelmans L, Van den Bulck J (2016) The use of media as a sleep aid in adults. Behav Sleep Med 14(2):121–133 25313639 10.1080/15402002.2014.963582

[23] Fahad LG, Tahir SF (2021) Activity recognition and anomaly detection in smart homes. Neurocomputing 423:362–372

[24] Frank E, Trigg L, Holmes G, Witten IH (2000) Naive bayes for regression. Mach Learn 41(1):5–25

[25] Howedi A, Lotfi A, Pourabdollah A (2020) A multi-scale fuzzy entropy measure for anomaly detection in activities of daily living. pp 1–8. ACM 10.3390/e22080845 PMC7517444 33286616

[26] Hsu M-F, Lee K-Y, Lin T-C, Liu W-T, Ho S-C (2021) Subjective sleep quality and association with depression syndrome, chronic diseases and health-related physical fitness in the middle-aged and elderly. BMC Publ Health 21:1–9 10.1186/s12889-021-10206-z PMC7816352 33468101

[27] Issa ME, Helmi AM, Al-Qaness MAA, Dahou A, Abd Elaziz M, Damaševičius R (2022) Human activity recognition based on embedded sensor data fusion for the internet of healthcare things. In: Healthcare, vol 10, p 1084. MDPI 10.3390/healthcare10061084 PMC9222808 35742136

[28] Jeyanthi A, Visumathi J, Genitha CH (2024) Enhanced two-stream Bayesian hyper parameter optimized 3d-CNN inception-v3 based drop-convlstm2d deep learning model for human action recognition. Inf Technol Control 53(1):53–70

[29] Kruijswijk J, van Emden R, Parvinen P, Kaptein M (2020) Streamingbandit: experimenting with bandit policies. J Stat Softw 94:1–47

[30] Masciadri A, Scarantino C, Comai S, Salice F (2019) Understanding home inactivity for human behavior anomaly detection, pp 90–95. ACM

[31] Mikolov T, Karafiát M, Burget L, Cernockỳ J, Khudanpur S (2010) Recurrent neural network based language model. In: Interspeech, vol 2, pp 1045–1048. Makuhari

[32] Moura I, Teles A, Coutinho L, Silva F (2022) Towards identifying context-enriched multimodal behavioral patterns for digital phenotyping of human behaviors. Future Gener Comput Syst 131:227–239

[33] Nazerfard E, Atashgahy Z, Nadali A (2021) Abnormal activity detection for the elderly people using convLSTM autoencoder

[34] Ng R, Sutradhar R, Yao Z, Wodchis WP, Rosella LC (2020) Smoking, drinking, diet and physical activity–modifiable lifestyle risk factors and their associations with age to first chronic disease. Int J Epidemiol 49(1):113–130 31329872 10.1093/ije/dyz078 PMC7124486

[35] Ordónez FJ, De Toledo P, Sanchis A (2013) Activity recognition using hybrid generative/discriminative models on home environments using binary sensors. Sensors 13(5):5460–5477 23615583 10.3390/s130505460 PMC3690009

[36] World Health Organization et al (2021) Decade of healthy ageing: baseline report. World Health Organization

[37] Parvin P, Chessa S, Kaptein M, Paternò F (2019) Personalized real-time anomaly detection and health feedback for older adults. J Ambient Intell Smart Environ 11(5):453–469

[38] Pashmdarfard M, Azad A (2020) Assessment tools to evaluate activities of daily living (ADL) and instrumental activities of daily living (IADL) in older adults: a systematic review. Med J Islam Repub Iran 34:33 32617272 10.34171/mjiri.34.33 PMC7320974

[39] Peterson LE (2009) K-nearest neighbor. Scholarpedia 4(2):1883

[40] Powers DMW (2020) Evaluation: from precision, recall and f-measure to roc, informedness, markedness and correlation. arXiv:2010.16061

[41] de Carvalho Santos SGT, de Barros RSM (2020) Online AdaBoost-based methods for multiclass problems. Artif Intell Rev 53(2):1293–1322

[42] Schmidt H, Mah CL, Cook B, Hoang S, Taylor E, Blacksher E, Goldberg DS, Novick L, Aspradaki AA, Tzoutzas I et al (2016) Chronic disease prevention and health promotion. Public health ethics: Cases spanning the globe, pp 137–176

[43] Singh D, Merdivan E, Hanke S, Kropf J, Geist M, Holzinger A (2017) Convolutional and recurrent neural networks for activity recognition in smart environment, pp 194–205

[44] Sparapani RA, Teng BQ, Hilbrands J, Pipkorn R, Feuling MB, Goday PS (2022) Novel pediatric height outlier detection methodology for electronic health records via machine learning with monotonic Bayesian additive regression trees. J Pediatr Gastroenterol Nutr 75(2):210–214 35641892 10.1097/MPG.0000000000003492

[45] Sprint G, Cook D, Fritz R, Schmitter-Edgecombe M (2016) Detecting health and behavior change by analyzing smart home sensor data, pp 1–3. IEEE

[46] Suganthan PN, Katuwal R (2021) On the origins of randomization-based feedforward neural networks. Appl Soft Comput 105:107239

[47] Tapia EM, Intille SS, Larson K (2004) Activity recognition in the home using simple and ubiquitous sensors. In: International conference on pervasive computing, pp 158–175. Springer

[48] Van Houdt G, Mosquera C, Nápoles G (2020) A review on the long short-term memory model. Artif Intell Rev 53(8):5929–5955

[49] van Kasteren TLM, Englebienne G, Kröse BJA (2011) Human activity recognition from wireless sensor network data: benchmark and software. In: Activity recognition in pervasive intelligent environments, pp 165–186. Springer

[50] Vaswani A, Shazeer N, Parmar N, Uszkoreit J, Jones L, Gomez AN, Kaiser Ł, Polosukhin I (2017) Attention is all you need. Advances in neural information processing systems, vol 30

[51] Wang R, Chen F, Chen Z, Li T, Harari G, Tignor S, Zhou X, Ben-Zeev D, Campbell AT (2017) Studentlife: using smartphones to assess mental health and academic performance of college students. Mobile health: Sensors, analytic methods, and applications, pp 7–33

[52] Yahaya SW, Lotfi A, Mahmud M (2019) A consensus novelty detection ensemble approach for anomaly detection in activities of daily living. Appl Soft Comput 83

[53] Yasugaki S, Okamura H, Kaneko A, Hayashi Y (2023) Bidirectional relationship between sleep and depression. Neurosci Res 10.1016/j.neures.2023.04.006 37116584

