# Supplementary material for: Smart home-assisted anomaly detection system for older adults: a deep learning approach with a comprehensive set of daily activities
Source: Med Biol Eng Comput. 2025 Jan 31;63(6):1821–35. doi: 10.1007/s11517-025-03308-y (PMC12106144; doi:10.1007/s11517-025-03308-y)
Supplement: Supplementary file 1 — (pdf 1047 KB) [file 11517_2025_3308_MOESM1_ESM.pdf]

# Supplementary Materials

**Smart Home-Assisted Anomaly Detection System for Older Adults: A Deep Learning Approach with a Comprehensive Set of Daily Activities**

**Name of authors:**
Ander Cejudo[1,3],
Andoni Beristain[1,2,4]
Aitor Almeida[3]
Kristin Rebescher[1]
Cristina Martín[1,2,3]
Ivan Macía[1,2,4]

**Corresponding author:**
Ander Cejudo[1,3] (acejudo@vicomtech.org)

**Affiliations:**
[1]Fundación Vicomtech, Basque Research and Technology Alliance (BRTA), Mikeletegi 57, 20009 Donostia-San Sebastián (Spain
[2]e-Health Department, Biodonostia Health Research Institute, Paseo Dr Begiristain s/n 20014 San Sebastián, Spain
[3]Faculty of Engineering, University of Deusto, Avda. Universidades, 24, Bilbao 48007, Spain
[4]Computational Intelligence Group, Computer Science Faculty, University of the Basque Country, UPV/EHU

**Journal:**
Medical & Biological Engineering & Computing

This document extends the results and the discussion presented in the main manuscript for discovering activity patterns, next day prediction and anomaly detection.

# 1 Activity patterns

In this section complementary information is provided for the visualization of activity patterns. For that, subsection 1.1 explains the methods considered (extending the definitions of section 4 in the main manuscript) for clustering and visualizing activity patterns. In subsection 1.2, the results of applying these techniques are shown and discussed.

## 1.1 Methodology for the visualization of activity patterns

A manual inspection is presented in order to identify relationships between days and discover activity patterns and users' behaviours. Two days ($c$ and $l$) are related or similar if they correspond to the same user ($U_i$) and if these days are proximal, as shown in equation (1) (i.e. distance between two days lower than a threshold $\tau$).

$$d(D_{ic}, D_{il}) < \tau \tag{1}$$

The UMAP [3] function is used to reduce the dimensionality of daily data ($D_{ij}$) to a three-dimensional space $\mathbb{R}^3$, as shown in equation (2). We have also considered PCA [1] and T-SNE [6] as dimensionality reduction techniques but preliminary experiments indicated that UMAP prduced clearer groupings and better visualization of similarities.

$$\text{UMAP} : \mathbb{R}^t \rightarrow \mathbb{R}^3 \tag{2}$$

UMAP is a dimension reduction technique that facilitates the visualization of clustering patterns in high-dimensional data. This method is based on manifold learning techniques and topological data analysis. First, "simplices" are built which is a $k$ dimensional object created by taking $k + 1$ independent objects ($D_{ij}$ in this case). Multiple simplices are then combined to form topological spaces, effectively gluing them together along their faces. A low-dimensional representation is optimized to closely match the topological structure of the original space, using the cross-entropy function [4] as a measure.

Furthermore, each day ($D_{ij}$) has been described with the K-means [5] clustering algorithm. Combined with UMAP, each day represented in space is colored with the cluster assigned by the clustering method. K-means has as a parameter the number of clusters ($k$) and, initially, each centroid (i.e. the mean of all the points within the same cluster) is assigned randomly to a point. Then, for each point, (i.e. $D_{ij}$) the nearest centroid is assigned and the centroids are computed again after going through all the points. This is an iterative process that ends when there are no more changes in the cluster assignation.

## 1.2 Visualization of activity patterns

In this additional experiment, relationships between the registered days are explored by means of clustering and three different dimensionality reduction techniques (i.e. PCA, T-SNE and UMAP), resulting in a three dimensional plot. The objective of this experiment is to discover data patterns that could be modelled to learn different user

behaviours from the information provided by the whole set of activities. Data patterns can be found by applying the proposed clustering technique to group similar days with related activity dedications or by having the days of the same user grouped in space. We hypothesize that the same user will have the days close in space with different levels of variabilities (i.e. points grouped but closer or further than other users) and some overlapping points between users with similar behaviours.

For visualization, TensorBoard[2] was employed and each point represents a day of one of the users in the dataset as in equation (??). T-SNE has been applied using the default settings: a learning rate of 10, perplexity equal to 12 and supervise disabled. The number of neighbors used for the UMAP algorithm is 15 after varying it from 5 to 50. The days are colored either by the assigned cluster (see Fig. 1) or by user (see Fig. 2-4). Applying these dimensionality reduction techniques results in three new variables that incorporate most of the variability of the 41 activities considered in this paper. With this, the data can be depicted in a three-dimensional space and help discover relationships between days and between different groups or clusters. If the days corresponding to a given user are proximal to one another, that would indicate that the user has a low varying behaviour, showcasing the feasibility of training a model capable of predicting the next day of a user. If the days of a given user are distant, a high variability of user behaviour would be expected, making it more difficult to estimate the user's behaviour.

Fig. 1 shows the results of K-means applied to each day. The clustering method allowed us to separate four groups of data in the space, resulting in a silhouette score of 0.1872 [7]. Most of the days are located close in space indicating that these users may have a similar behaviour (with some variations) except for the dark blue group, which is distant from the rest of the groups. Fig. 2-4 have each day colored according to the user. Comparing the three dimensionality reduction algorithms, T-SNE and UMAP group the days of the same user close in space while PCA has a more disorganized outcome. T-SNE isolates more the users in space but UMAP is capable of showing similar behaviours (overlapping points between users). Thus, in Fig. 4 some users have their days located in proximal regions (e.g. dark blue, orange and purple) with low levels of variability (days are close) whereas others have a higher variability (e.g. dark green and pink). Although some users have more variability than others, all of them have a high percentage of the days in a specific region in space and some users share a similar behaviour (i.e. some of their days are very close in space).

As a conclusion, relationships between days have been found as well as patterns in the data that could be defined as different behaviours (see Table 1). Thus, we hypothesize that those behaviours could be effectively modelled with the information provided by the whole set of activities.

Overall, Fig. 4 serves as the starting point of our study as the days of some users in most of the cases are close in space and is what we propose as the definition of behaviour in an exploratory manner. In addition, a region shared by most of the users can be appreciated and, at the same time, this area is far from the common behavior of some users (e.g. orange user). For this reason, by training the algorithms at population level, we intend to avoid identifying these cases as anomalies, as even if not being a usual behaviour for the user, they still represent the normality at population level as

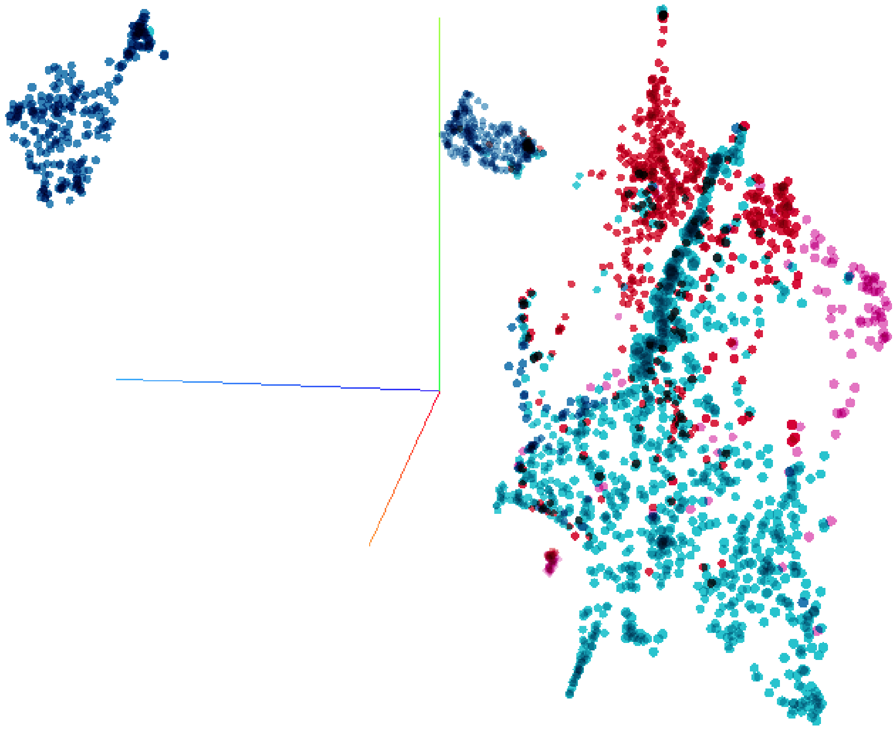

**Fig. 1** Result of users' days visualization in a three dimensional space being each point a day and each color 1 of the 4 groups assigned by the K-means clustering algorithm.

they could be related to, for example, weekends. Thus, the recurrent neural network model learns from all the users at once, as we hypothesize that, for example: if a given user never does exercise and one day starts doing it, that day will likely be marked as an anomaly by previous approaches. In our case, as we take advantage of deep learning models and all the data, the model will know that doing exercise with a certain duration is normal in the current population, so the likeliness of detecting an anomaly will be reduced, decreasing the false positives rate. The potential anomalies are those that are out of the cloud of dots in Fig. 4, for example, between the majority of orange and purple days.

In addition, the Fig. 4 helps in the selection of a p-value for the first anomaly detection step, as a population with a higher variability (i.e. young and healthy people) would require a higher p-value compared to the population of older adults in this dataset. Thus, the p-value depends on the population and must be decided with the caregivers, whether they want to be aware of any minimal change (i.e. low p-value) or just to be notified when a significant deviation occurs (i.e. high p-value).

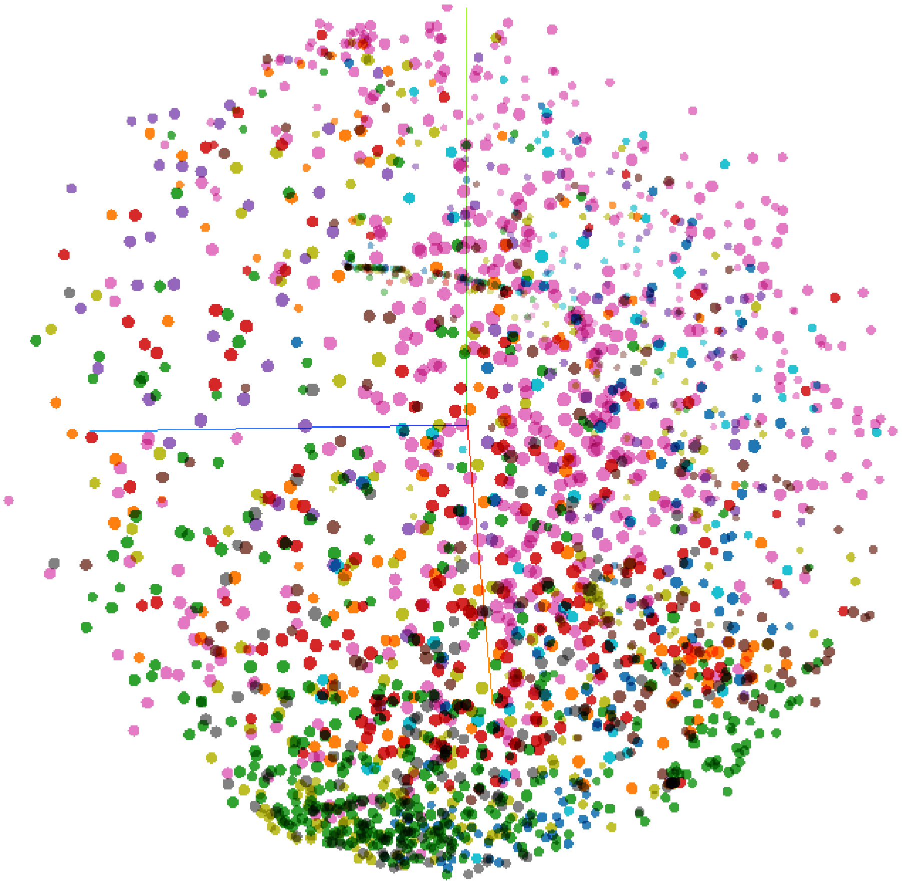

**Fig. 2** Result of plotting days colored by user in a three dimensional space with the PCA dimensionality reduction algorithm.

In Table 1 a 45.97% of the population is in "Cluster 3" where the time spent for sleeping is the highest along with the time spent for grooming and dressing. In "Cluster 0" and "Cluster 2" the time spent watching TV is considerable with over a 16% of mean daily dedication, but in "Cluster 2" the value for sleeping out of bed is more than a 19% of mean daily dedication, which can be related to watching TV. Finally, in "Cluster 1" days with a high mean daily dedication to entertaining guests have been grouped.

## 2 Next day prediction

This section extends section 5.2 which contains the results of the proposed models for next day prediction. Figure 5 depicts the different recurrent neural network (RNN)-based architectures used in this study.

**Table 1** Centroids of the four clusters obtained with the K-means clustering algorithm with a custom description (given looking at meaningful differences between activities), the count (i.e. number of days in the cluster) the percentage (i.e. relative percentage in number of days compared with other clusters) and the dedication for each activity (given in a daily percentage format, over 24 hours). The highest value per row has been marked.

| | **Cluster 0** | **Cluster 1** | **Cluster 2** | **Cluster 3** |
|---|---|---|---|---|
| Description | A lot of time working, dressing and watching TV. | A lot of time with guests and cooking breakfast. Half the day is for other activities (probably because of guests). | Low sleep in bed and much time sleeping out of bed and watching TV. | Long sleep in bed and much time spent in dressing, grooming, personal hygiene and bathe. Also eating breakfast and working in computer. |
| Count | 535 | 529 | 149 | **1032** |
| Percentage | 23.83 | 23.56 | 6.63 | **45.97** |
| Bathe | 0.28 | 0.24 | 0.62 | **1.64** |
| Bed_Toilet_Transition | 0.07 | **1.68** | 0.13 | 0.26 |
| Cook | 0.24 | 0.27 | 0.12 | **0.49** |
| Cook_Breakfast | 0.16 | **4.27** | 0.06 | 1.72 |
| Cook_Dinner | 0.28 | **0.70** | 0.68 | 0.07 |
| Cook_Lunch | 0.25 | **0.35** | 0.24 | 0.09 |
| Dress | 1.29 | 0.68 | 0.64 | **1.38** |
| Drink | 0.27 | 0.16 | 0.18 | **0.30** |
| Eat | **0.29** | 0.18 | 0.21 | 0.06 |
| Eat_Breakfast | 0.39 | 0.51 | 0.61 | **1.28** |
| Eat_Dinner | 0.22 | **0.64** | **0.59** | 0.08 |
| Eat_Lunch | 0.20 | **0.41** | 0.28 | 0.08 |
| Enter_home | 0.09 | 0.08 | 0.10 | **0.17** |
| Entertain_Guests | 8.70 | **27.38** | 11.91 | 14.78 |
| Evening_Meds | **0.09** | 0.04 | 0.07 | 0.07 |
| Exercise | 0.00 | 0.00 | 0.00 | 0.00 |
| Go_To_Sleep | 0.00 | 0.00 | 0.00 | 0.00 |
| Groom | 0.62 | 0.41 | 0.65 | **1.94** |
| Landry | **0.04** | 0.00 | 0.00 | 0.00 |
| Leave_Home | 0.10 | 0.10 | 0.10 | **0.22** |
| Morning_Meds | **0.12** | 0.04 | 0.07 | 0.05 |
| Nap | 0.01 | **0.07** | 0.00 | 0.00 |
| Personal_Hygiene | 2.58 | 1.26 | 1.33 | **4.28** |
| Phone | 0.77 | 0.39 | 0.53 | **1.11** |
| Read | 1.66 | 1.47 | **1.73** | 0.71 |
| Relax | 0.65 | 1.16 | 0.80 | **1.40** |
| Sleep | 31.92 | 29.61 | 28.14 | **33.08** |
| Sleep_Out_Of_Bed | 1.10 | 1.66 | **19.37** | 0.36 |
| Step_Out | 0.09 | 0.12 | **0.18** | 0.06 |
| Take_Medicine | **0.12** | 0.03 | 0.02 | 0.01 |
| Toilet | 0.67 | 0.63 | 0.80 | **0.91** |
| Wake_up | 0.00 | 0.00 | 0.00 | 0.00 |
| Wash_Breakfast_Dishes | 0.20 | 0.17 | 0.12 | **0.92** |
| Wash_Dinner_Dishes | 0.09 | 0.21 | **0.24** | 0.01 |
| Wash_Dishes | 0.37 | 0.37 | 0.62 | **1.19** |
| Wash_Lunch_Dishes | 0.07 | **0.10** | 0.04 | 0.04 |
| Watch_TV | **16.98** | 4.59 | 16.14 | 1.53 |
| Work | **1.24** | 0.18 | 0.16 | 0.10 |
| Work_At_Desk | 0.00 | **0.13** | 0.00 | 0.00 |
| Work_At_Table | 0.18 | 0.00 | 0.00 | **1.01** |
| Work_On_Computer | 0.71 | **4.14** | 2.30 | 3.77 |

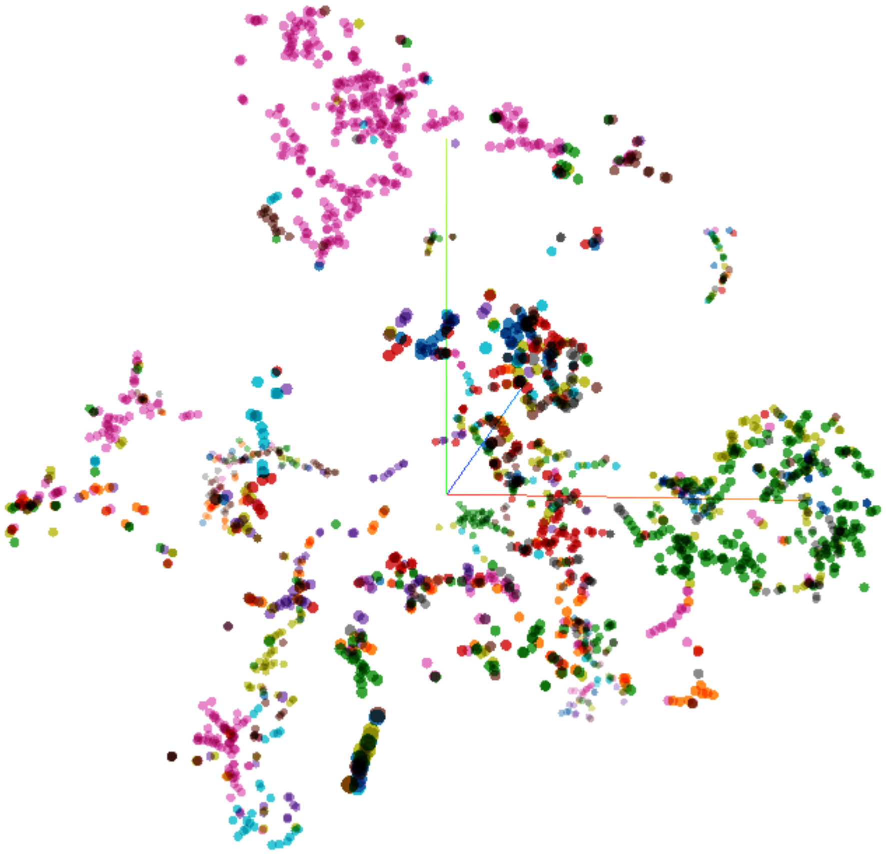

**Fig. 3** Result of plotting days colored by user in a three dimensional space with he T-SNE dimensionality reduction algorithm.

Taking the AttentionRNN as the best model for next day prediction, a visual inspection has been carried out. The one-day prediction output allows to subtract the actual and predicted value, given that a day dedication percentage for each activity is estimated. For that reason, we have selected two meaningful activities in the daily routine of a user (i.e. sleep and watch TV) and we have depicted the difference between the actual and the predicted values over time for each the training and test sets in Fig. 6-9.

Fig. 6 and Fig. 7 depict a comparison between the train and test sets for sleep activity. For the training set, the model shows a good fit after training, being able to better adapt to a considerable decrease in the sleep dedication rather than an unexpected increase with values over 40% in daily dedication percentage. In addition, the regularization effect is noticeable as the predicted value is mostly in between the previous, current and next day value, avoiding overfitting and capturing either the

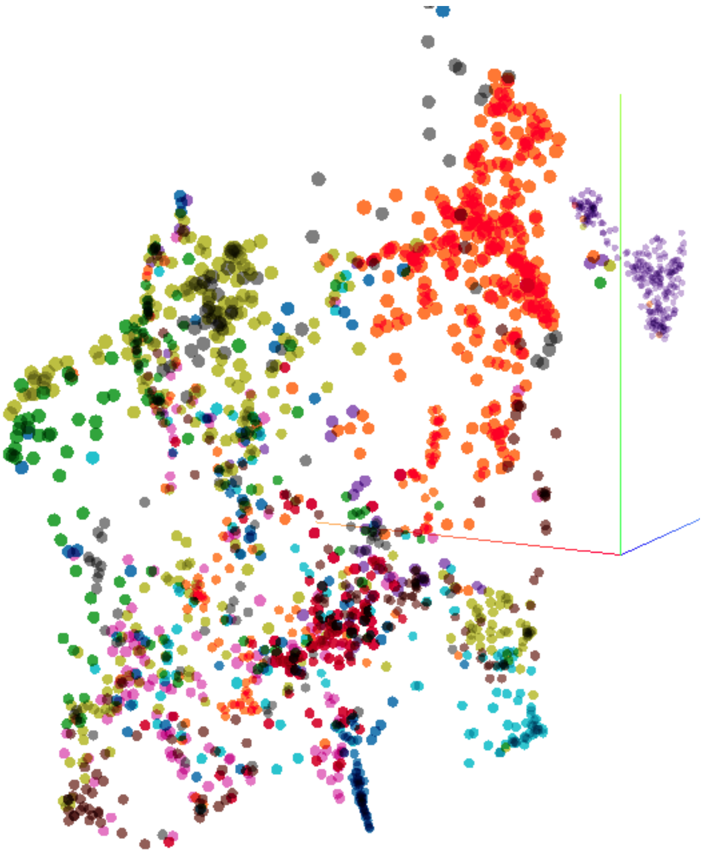

**Fig. 4** Result of plotting days colored by user in a three dimensional space with the UMAP dimensionality reduction algorithm.

increase or decrease in the dedication of that day. Notice that the predicted activities present lower variability than the actual activities (both using the training and test sets), which means that our approach is forecasting not single values but behaviour changes. That is, if the actual value is higher than the previous one in the time series, the predicted values is also going to be higher in most of the cases.

In addition, Fig. 8 and 9 show a comparison between the train and test sets for "Watch_TV" activity . For this activity, the model has shown a better fit capability compared to the "Sleep" activity in both the training and the test sets. For the days when the dedication to watching TV is close to 0 is where the model seems to fail in many cases, as it predicts a higher value. Furthermore, the model gets closer to lower values rather than to higher values. Both behaviours are shared across all the activities, as regularization has been applied to improve the performance of next day prediction and extreme values such as 0% of daily dedication are not predicted. As the objective is not to predict single activities but to capture the overall trend and model the different behaviours that people may have for anomaly detection, a common

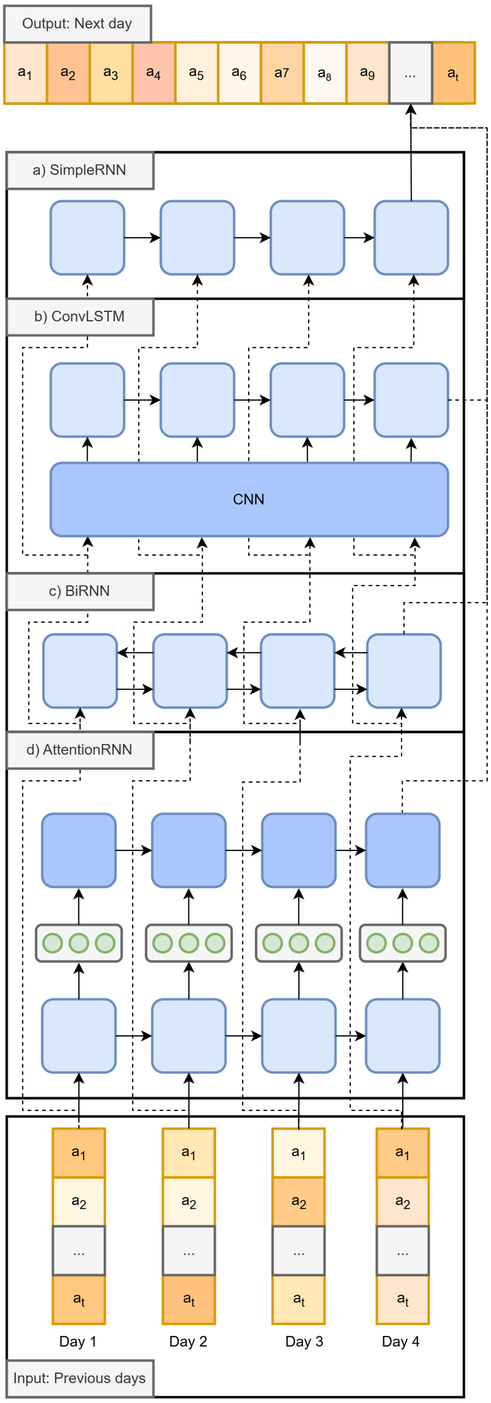


**Fig. 5** Representation of different recurrent neural network architecutres employed in this study: (a) RNN, (b) ConvLSTM, (c) BiRNN and (d) AttentionRNN. For each experiment, one of these architectures is selected independently and the number of previous days in the input ($w$) is tuned to achieve the best next day prediction performance. In this example, four days are inputted and the fifth day is predicted. Note that the algorithms are not combined, but share the same input and output format.

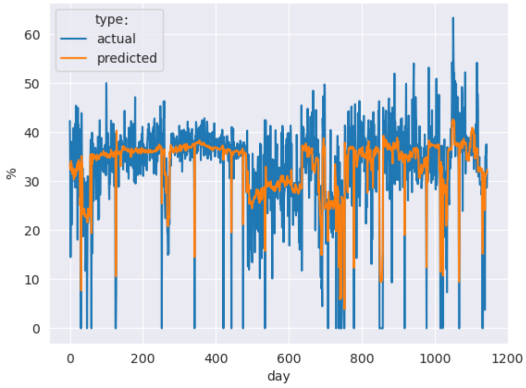


**Fig. 6** Fit in the training set by the AttentionRNN for the "Sleep" activity, with the activity day dedication percentage in the y axis and window size set to 15.

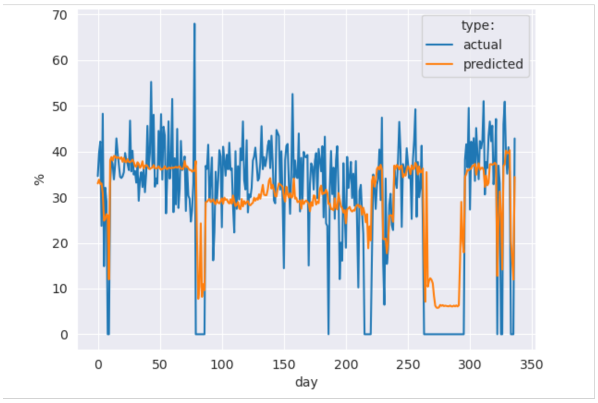


**Fig. 7** Fit in the test set by the AttentionRNN for the "Sleep" activity, with the activity day dedication percentage in the y axis and window size set to 15.

error of 2-3% when the dedication has been nearly 0% will be considered statistically common and will not be marked as an anomaly.

Empirically, the model evaluation and the depicted activity dedications have shown the capabilities of the model for next day estimation of some activities. In addition, the weaknesses of the model are captured and the overall tendency seems to be well represented. The hyperparameters used for training the selected deep learning models are shown in Table 2. The loss of these models on the test set throughout the training epochs is shown in Fig. 10. The hyperparameters for each model are presented in Table 2. Early stopping is set to 100 epochs. In Fig. 10, AttentionRNN is the model that achieves the lowest loss score and continues to decrease the loss as the number of epochs increases. For the RNN and ConvLSTM models, the loss starts to increase after 150 epochs, whereas the Neural_Net model shows a consistently low and stable loss. BiRNN performs similarly to AttentionRNN but with significant variations in the loss values.

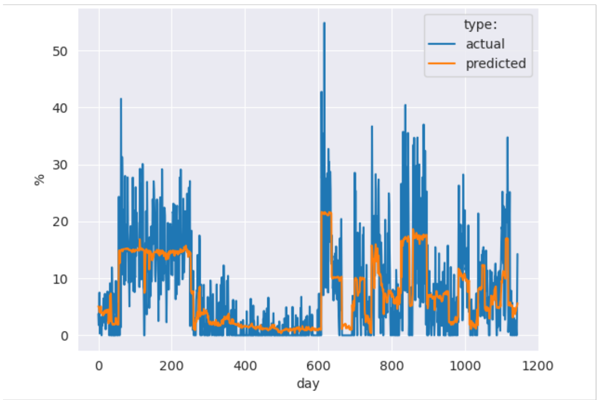


**Fig. 8** Fit in the training set by the AttentionRNN for the "Watch_TV" activity, with the activity day dedication percentage in the y axis and window size set to 15.

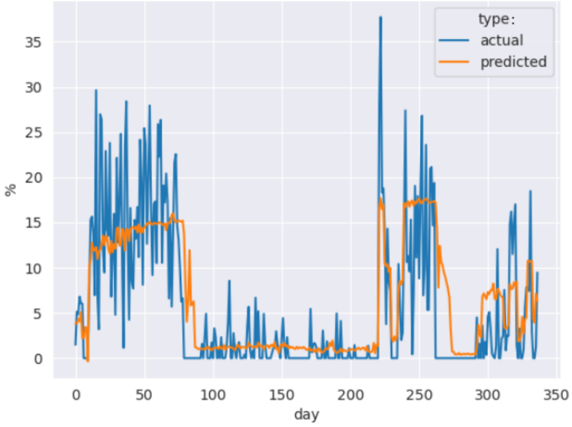


**Fig. 9** Fit in the test set of the AttentionRNN for the "Watch_TV" activity, with the activity day dedication percentage in the y axis and window size set to 15.

At the same time, the proposed model has been designed to be trainable as new data is gathered, adapting to possible behaviour changes of the users. For that, reason, if the users are modelled and a behaviour change is being produced in any of them (due to any health event), the likelihood of detecting anomalies will be increased but if the new behaviour is long-term, the model can be able to adapt to the new situation.

Among the metrics used for model performance evaluation, Mean Squared Error (MSE) is considered the most relevant, as it penalizes large errors that could be marked as anomalies. Therefore, selecting the model with the lowest MSE is crucial for accurate behavior modeling. A lower MSE score indicates a better understanding of the various behaviors represented in the dataset. This ensures that significant deviations detected by the double-step anomaly detection algorithm are based on deviations from the typical behavior of a specific user as interpreted by the selected model. However, if the

**Table 2** Hyperparameters used for training the selected deep learning models: Neural_Net, RNN, ConvLSTM, BiRNN and AttentionRNN. The window size, learning rate and batch size are shared for all the models, whereas the remaining hyperparameters have been optimized with Optuna for each window size.

| Hyperparameter | Neural_Net | RNN | ConvLSTM | BiRNN | AttentionRNN |
|---|---|---|---|---|---|
| Window | 1 | 15 | 15 | 15 | 15 |
| Learning rate | 0.1 | 0.1 | 0.1 | 0.1 | 0.1 |
| Batch size | 128 | 128 | 128 | 128 | 128 |
| # CNN layers | – | – | 1 | – | – |
| # CNN filters | – | – | 13 | – | – |
| CNN kernel size | – | – | 26 | – | – |
| # LSTM layers | – | 1 | 2 | 1 | 2 |
| # LSTM neurons | – | 109 | 71 | 161 | 61 |
| # FF layers | 2 | 2 | 1 | 1 | 3 |
| # FF neurons | 53 | 53 | 43 | 53 | 13 |
| Dropout | 0.1 | 0.1 | 0.2 | 0.2 | 0.04 |

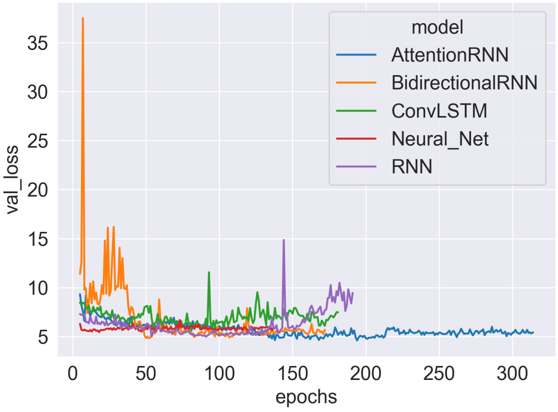


**Fig. 10** Comparison of the loss (MSE) throughout the training epochs for the proposed deep learning models: Neural_Net, RNN, ConvLSTM, BiRNN and AttentionRNN.

model's understanding of behavior is insufficient, significant deviations or patterns may be incorrectly flagged as anomalies due to improper modeling. While this limitation could also affect the proposed approach, selecting the best-performing model based on MSE helps mitigate this issue. Future research could explore alternative architectures or model variants to enhance next day prediction performance and, consequently, improve behavior modeling in the target population.

# 3 Anomaly detection

This section extends the results reported in subsection 5.2. As seen in Fig. 4, some data points of different users are further from the typical behaviour of the user (i.e. cloud of points in space) and are not close to the behaviour of other users. The proposed

methodology is expected to mark those days as anomalies and to offer an interpretable explanation about the deviated activities and the dedication limit surpassed.

Fig. 11 shows the error distribution of the proposed AttentionRNN. The model commonly produces errors ranging between 0.6 and 0.8 in terms of MAE. The mean value of all errors is deemed representative after passing a t-test (p-value $< 0.05$) and rejecting the null hypothesis. This outcome suggests that no bias has been detected in the training of the model.

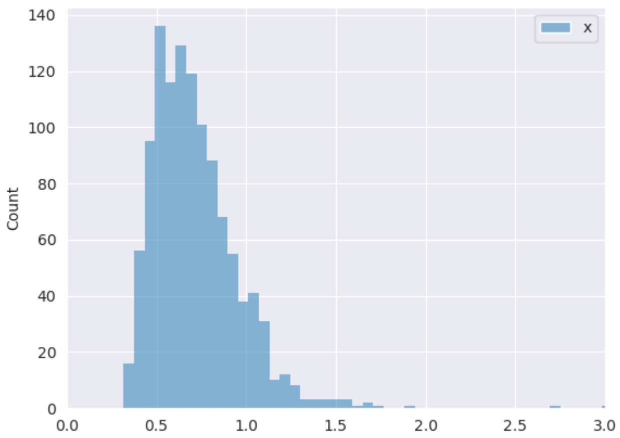


**Fig. 11** Histogram of the errors produced in the training set by the AttentionRNN for next day prediction in terms of mean absolute error (MAE).

The way that the model has been trained, when detecting an anomaly in the first step, the user's trajectory is considered as input but maintaining a general knowledge in the population, so transitions from the user's behaviour to another close behaviour may not be considered as abnormal which may also be biased by the day of the week (e.g weekend). In the second step, we take into account the dedication of each activity at population level in order to reduce the number of false positives by not considering as an anomaly a day that has no significant deviated activity. This step considers that there is a dependency between the dedication of the activity, as a significant change in any of the activities would affect the others. The first step has been modelled with a normal distribution as we have seen in Fig. 11 how the errors of the model behave, but for the second step, as we do not know the distribution behind each of the activities and it is conditioned to the population of the study, a boxplot outlier detection has been considered. When the mean value of all errors, determined through a t-test (p-value $< 0.05$) and rejection of the null hypothesis, remains representative as in this case, it suggests that no bias has been detected during the model's training. In addition, when considering all the data points from all users (see Fig. 6 and 8), a correct modelling of the tendency is shown for the different users, which also shows the absence of any bias in the training process.

The output of the double-step anomaly detection system is designed to be easily interpretable (see Table ??). For each user, it highlights the days identified as anomalies and specifies the activities that exceeded the expected dedication. These deviations are evaluated not only against the user's common behavior, as learned by the next day prediction model, but also in comparison to population-level thresholds based on the first and third quartiles in boxplot analysis. The output format, as illustrated in Table ??, aligns with approaches used in previous works [2]. Additionally, for each activity, a detailed comparison of actual versus predicted values is provided, as shown in Fig. 6-9. This enables clinicians to assess whether the detected deviations accurately reflect meaningful changes.

A manual inspection of the developed model's capabilities for next day activity prediction has shown a good fit although the distance between the predicted value and the actual is significant in some cases, but the objective is to learn different behaviours and not to get a perfect fit for each activity. Consequently, this model has been used for anomaly detection combining it with statistical methods based on the error produced by the model along with the expected error probability in the detected anomalies. The proposed double-step anomaly detection system is capable of detecting anomalies on unseen data for days with an unexpected user behaviour and the second step of the proposed method provides interpretability as activities deviated from the common dedication range can be seen. It is also remarkable that the same proportion of anomalies has been detected in both the train and the test sets.

Regarding the anomalies that have been detected in the training step and have been used to train the model, we hypothesize that this amount of reported anomalies is due to the selected p-value, that doesn't adjust to the high variability of daily routines of some of the users in Fig. 4 and a higher p-value could have been more convenient, but that depends on the use case and the needs of the caregivers. In any case, in the training process the model tries to adjust to the majority of the examples and anomalies are defined like a minority set of examples because if anomalies start to increase, assuming that the model is further trained, the abnormal days will become the normality since the overall error is reduced in next day prediction.

# References


[1] Hervé Abdi and Lynne J Williams. Principal component analysis. *Wiley interdisciplinary reviews: computational statistics*, 2(4):433–459, 2010.

[2] Khaled A Alaghbari, Mohamad Hanif Md Saad, Aini Hussain, and Muhammad Raisul Alam. Activities recognition, anomaly detection and next activity prediction based on neural networks in smart homes. *IEEE Access*, 10:28219–28232, 2022.

[3] Leland McInnes, John Healy, and James Melville. Umap: Uniform manifold approximation and projection for dimension reduction. *arXiv preprint arXiv:1802.03426*, 2018.

[4] Leland McInnes, John Healy, Nathaniel Saul, and Lukas Grossberger. Umap: Uniform manifold approximation and projection. *The Journal of Open Source Software*, 3(29):861, 2018.

[5] Kristina P Sinaga and Miin-Shen Yang. Unsupervised k-means clustering algorithm. *IEEE access*, 8:80716–80727, 2020.

[6] Laurens van der Maaten and Geoffrey Hinton. Viualizing data using t-sne. *Journal of Machine Learning Research*, 9:2579–2605, 11 2008.

[7] Fei Wang, Hector-Hugo Franco-Penya, John Kelleher, John Pugh, and Robert Ross. An analysis of the application of simplified silhouette to the evaluation of k-means clustering validity. 07 2017.
